# Lung eQTLs to Help Reveal the Molecular Underpinnings of Asthma

**DOI:** 10.1371/journal.pgen.1003029

**Published:** 2012-11-29

**Authors:** Ke Hao, Yohan Bossé, David C. Nickle, Peter D. Paré, Dirkje S. Postma, Michel Laviolette, Andrew Sandford, Tillie L. Hackett, Denise Daley, James C. Hogg, W. Mark Elliott, Christian Couture, Maxime Lamontagne, Corry-Anke Brandsma, Maarten van den Berge, Gerard Koppelman, Alise S. Reicin, Donald W. Nicholson, Vladislav Malkov, Jonathan M. Derry, Christine Suver, Jeffrey A. Tsou, Amit Kulkarni, Chunsheng Zhang, Rupert Vessey, Greg J. Opiteck, Sean P. Curtis, Wim Timens, Don D. Sin

**Affiliations:** 1 Merck Research Laboratories, Boston, Massachusetts, United States of America; 2 Merck, Rahway, New Jersey, United States of America; 3 Genetics, Rosetta Inpharmatics, Merck, Seattle, Washington, United States of America; 4 Department of Molecular Medicine, Laval University, Québec City, Canada; 5 Institut Universitaire de Cardiologie et de Pneumologie de Québec, Laval University, Québec City, Canada; 6 The University of British Columbia James Hogg Research Laboratory, St Paul's Hospital, Vancouver, Canada; 7 Respiratory Division, Department of Medicine, University of British Columbia, Vancouver, Canada; 8 Department of Pulmonology, University Medical Center Groningen, GRIAC Research Institute, University of Groningen, Groningen, The Netherlands; 9 Department of Pathology and Laboratory Medicine, University of British Columbia, Vancouver, Canada; 10 Department of Pathology and Medical Biology, University Medical Center Groningen, GRIAC Research Institute, University of Groningen, Groningen, The Netherlands; 11 Department of Pediatric Pulmonology, University Medical Center Groningen, University of Groningen, Groningen, The Netherlands; 12 Sage Bionetworks, Seattle, Washington, United States of America; Dartmouth College, United States of America

## Abstract

Genome-wide association studies (GWAS) have identified loci reproducibly associated with pulmonary diseases; however, the molecular mechanism underlying these associations are largely unknown. The objectives of this study were to discover genetic variants affecting gene expression in human lung tissue, to refine susceptibility loci for asthma identified in GWAS studies, and to use the genetics of gene expression and network analyses to find key molecular drivers of asthma. We performed a genome-wide search for expression quantitative trait loci (eQTL) in 1,111 human lung samples. The lung eQTL dataset was then used to inform asthma genetic studies reported in the literature. The top ranked lung eQTLs were integrated with the GWAS on asthma reported by the GABRIEL consortium to generate a Bayesian gene expression network for discovery of novel molecular pathways underpinning asthma. We detected 17,178 *cis-* and 593 *trans-* lung eQTLs, which can be used to explore the functional consequences of loci associated with lung diseases and traits. Some strong eQTLs are also asthma susceptibility loci. For example, rs3859192 on chr17q21 is robustly associated with the mRNA levels of *GSDMA* (*P* = 3.55×10^−151^). The genetic-gene expression network identified the SOCS3 pathway as one of the key drivers of asthma. The eQTLs and gene networks identified in this study are powerful tools for elucidating the causal mechanisms underlying pulmonary disease. This data resource offers much-needed support to pinpoint the causal genes and characterize the molecular function of gene variants associated with lung diseases.

## Author Summary

Recent genome-wide association studies (GWAS) have identified genetic variants associated with lung diseases. The challenge now is to find the causal genes in GWAS–nominated chromosomal regions and to characterize the molecular function of disease-associated genetic variants. In this paper, we describe an international effort to systematically capture the genetic architecture of gene expression regulation in human lung. By studying lung specimens from 1,111 individuals of European ancestry, we found a large number of genetic variants affecting gene expression in the lung, or lung expression quantitative trait loci (eQTL). These lung eQTLs will serve as an important resource to aid in the understanding of the molecular underpinnings of lung biology and its disruption in disease. To demonstrate the utility of this lung eQTL dataset, we integrated our data with previous genetic studies on asthma. Through integrative techniques, we identified causal variants and genes in GWAS–nominated loci and found key molecular drivers for asthma. We feel that sharing our lung eQTLs dataset with the scientific community will leverage the impact of previous large-scale GWAS on lung diseases and function by providing much needed functional information to understand the molecular changes introduced by the susceptibility genetic variants.

## Introduction

Recent genome-wide association studies (GWAS) have identified loci that harbor susceptibility genes for asthma and other pulmonary conditions [1]–[16]. Many of the genes at these loci have unknown function and have not previously been considered biologically plausible candidates for disease pathogenesis. Extensive linkage disequilibrium (LD) within these loci makes it difficult to identify the actual susceptibility genes, let alone which genetic variants are responsible for altered expression or function of their protein products. Moreover, the associated polymorphisms can only explain a relatively small proportion of the variability of the phenotype in the population [9] and of its heritability [17].

Integrative genomics is a promising new approach to identify true causal genes and variants. By using gene expression as a phenotype and examining how DNA polymorphisms contribute to both gene expression (expression quantitative trait loci – eQTLs) and disease phenotype, true causal relationships can be discovered [18]–[20]. In the present study we performed genome-wide genotyping and lung-specific gene expression on a large dataset of lung tissue (1,111 human subjects) to explore effects of genetic variation on gene expression and their joint relationship to asthma. Although the lung tissue came from a heterogeneous group of subjects, the discovery of lung tissue eSNPs could elucidate the causal molecular pathways in a variety of pulmonary disorders.

## Results

### Demographic characteristics of study participants

**Figure 1:**
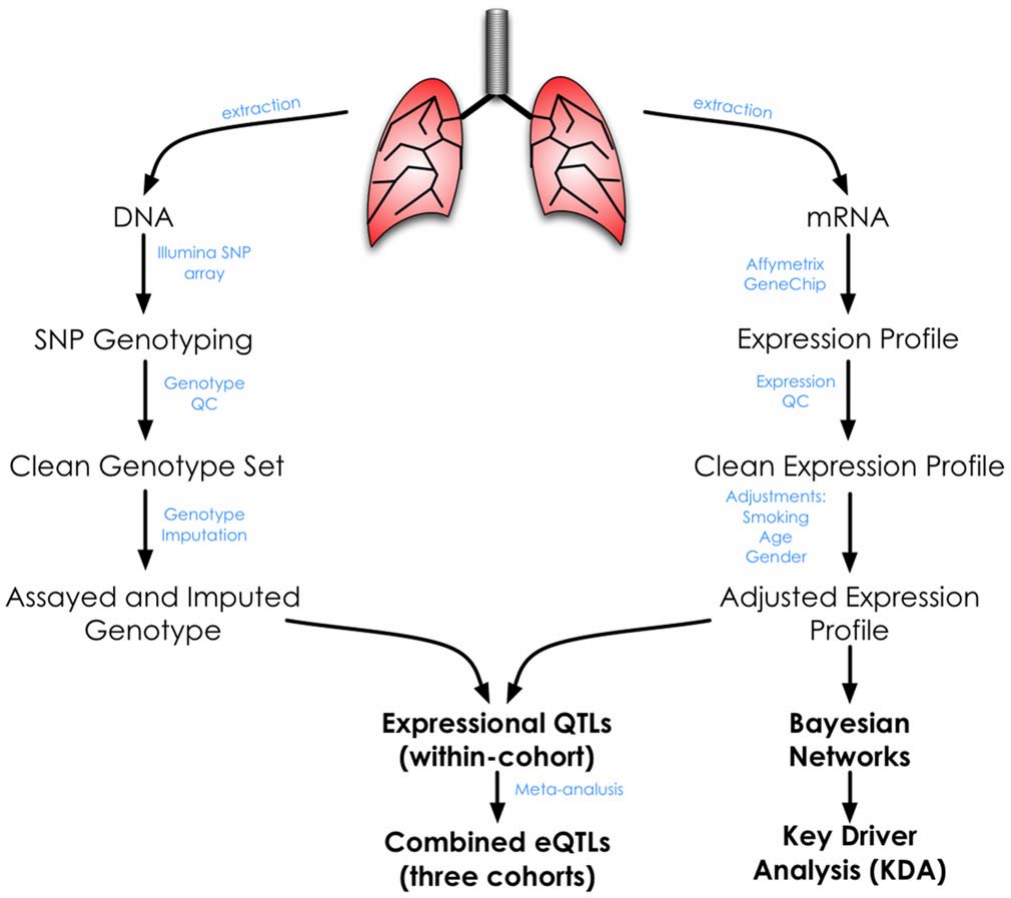
Overall study design and analysis workflow.

Demographic and clinical data for the 1,111 patients who passed clinical, genotyping and gene expression quality control assessments are summarized in Table 1 according to the three sites of recruitment, Laval University, University of British Columbia, and University of Groningen (henceforth referred to as Laval, UBC, and Groningen, respectively). The majority of the subjects were smokers or former-smokers. There were some differences in the clinical characteristics across the sites including age, lung function and smoking status. To account for this heterogeneity between sites, we performed a meta-analysis rather than a pooled analysis for eQTL discovery. The overall analysis workflow is illustrated in Figure 1.

**Table 1:** Demographic and clinical characteristics of study cohort.

	Lavaln = 409	Groningenn = 363	UBCn = 339
**Quantitative traits**, mean ± SD			
Age (years) *	63.3±10.0	51.8±15.2	60.0±14.3
PreBD FEV_1_ % predicted *	80.5±18.9	60.5±30.0	80.4±22.4
PreBD FVC % predicted *	89.8±16.1	75.0±26.5	88.9±19.7
**Categorical traits, %**			
Male	56.0	53.6	53.4
Smoking Status *			
Non-smoker	8.7	28.6	8.8
Ex-smoker	69.0	54.9	56.8
Current-smoker	22.2	16.5	34.4

PreBD, before bronchodilator use; FEV_1_, forced expiratory volume in one second; FVC, forced vital capacity.

* significantly different among groups at p<0.01 level (Kruskal Wallis and χ^2^ tests were applied to quantitative and categorical traits, respectively).

### Genome-wide association analysis for expression traits (eQTL)

We identified *cis*- and *trans*-acting eQTLs using established methods [19]. For simplicity we assumed that each transcript could have at most one *cis*-eQTL. We considered all association signals from SNPs within 1 Mb up and downstream, of the transcription probeset as a single *cis*-eQTL. *Trans*-eQTLs were defined as association signals from SNPs located greater than 1 Mb from the probeset. The eSNP was identified as the SNP that was most significantly associated (lowest *P* value) with the expression trait. A summary of the eQTLs identified at 10% false discovery rate (FDR) in the three cohorts as well as the meta-analysis are reported in Table 2. There were variations in the numbers of eQTLs across the cohorts. For example, we identified 10,630, 5,655 and 7,953 *cis*-eQTLs in the Laval, Groningen and UBC cohorts, respectively. There were also variations in the number of detectable *trans*-eQTLs (Table 2). The *P* values reported for the *trans*-eQTLs are lower than those for *cis*-eQTLs, owing to the higher statistical threshold required to achieve statistical significance. In general, a single SNP (eQTL peak) explained between 10 and 20% of the transcript's expression variance for *cis*- and *trans*-eQTLs, respectively (Table S1). However 6.8% of the *cis* acting SNPs explained more than 30% of the variance in the transcript and 15.3% of the *trans* acting SNPs explained more than 30% of a transcript's variance (Figure S1).

**Table 2:** Summary of expression QTLs detected at 10% false discovery rate.

	Laval	Groningen	UBC	Meta-Analysis
*cis*-eQTL *P* value cutoff	4.0e−05	2.3e−05	2.8e−05	5.9e−05
*trans*-eQTL *P* value cutoff	4.9e−10	1.2e−10	1.6e−10	7.0e−10
number of *cis*-eQTLs	10,630	5,655	7,953	17,049
number of *trans*-eQTLs	130	96	108	534

Further, we investigated the consistency of the eQTLs derived from the cohorts. Rather than simply comparing the existence of eQTLs, we adopted replication criteria similar to GWAS. A successful replication was defined as an eQTL in which the relationship between the same SNP and gene expression (with the same sign) was observed in more than one cohort at a *P* of ≤1×10^−4^. Using this criterion, 54.5% of the Laval *cis*-eQTLs were replicated in at least one other cohort and 79.5% of the Laval *trans*-eQTLs were replicated in another cohort. With respect to the Groningen eQTLs, the replication rates of the *cis*- and *trans*-eQTLs were 72.3% and 86.0%, respectively. The replication rates of the UBC *cis*- and *trans*-eQTLs were 65.4% and 90.5%, respectively. The eQTLs with higher R^2^values (variance of gene expression level explained) were more likely to be replicated than those with lower R^2^ values. There was a higher replication rate with *trans*-eQTLs than with *cis*-eQTLs.

The meta-analysis revealed many more eQTLs owing to the increased sample size (Table 2). We identified 17,049 *cis*-eQTLs and 534 *trans*-eQTL and the details of all eQTLs are listed in Table S2. 68.7% of these *cis*-eQTLs and 31.7% of *trans*-eQTLs were identified in at least one cohort, suggesting that the meta-analysis detected many additional eQTLs. The ‘non-combinability’ among cohorts is quantified in terms of Cochran's Q and the τ statistics (Table S2). Cochran's Q, was calculated as the weighted sum of squared differences among individual study effects and the pooled effect across studies, with the weights proportional to the inverse of variance of the eQTL effect. Q follows a chi-square statistic with k-1 degrees of freedom (k is the number of studies), and by these means, we calibrated the p value (termed Q.pvalue). We detected modest degrees of heterogeneity among the three cohorts. 16% of the QTLs had Q.pvalue<0.05 and 2% of the eQTLs showed Q.pvalue<0.001. We also present the τ statistic, which is the moment-based estimate of the between-study variance and is not dependent on the number of studies. Both the fixed effect and random effect meta-analysis results are presented in Table S2, and we used the fix effect results in the downstream analysis. We previously showed that the number of eQTLs detectable at a certain FDR was related directly to the sample size (i.e. statistical power) [21]. The meta-analysis yielded a greater relative increase in the number of *trans* activating eQTLs than of *cis*-eQTLs, suggesting that sample size is particularly important for detecting *trans*-eQTLs. Among the 51,627 non-control probesets on our array, 33.0% showed a *cis*-eQTL. 74.0%, 64.4%, 56.4% of the 51,627 probesets were “present” in at least 20%, 50%, and 80% of the tissue samples. Using these three cutoffs, 40.2%, 42.5%, and 43.4% of the “present” transcripts were found to be controlled by cis-eSNPs, respectively. Accordingly we believe that we have identified the majority of the strong lung cis-eQTLs.

**Figure 2:**
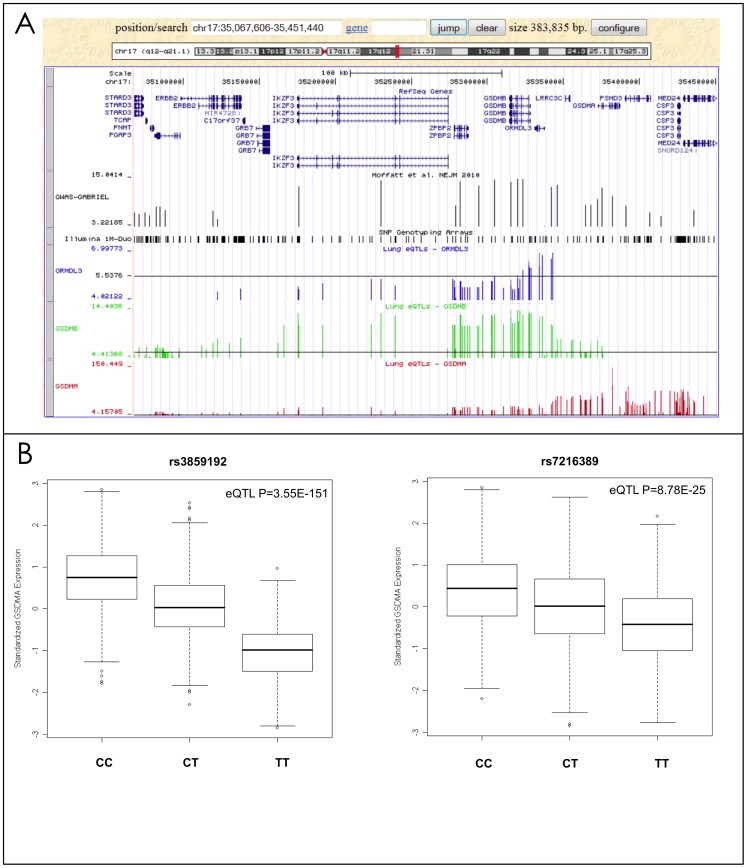
Lung eQTLs found on chromosome 17q21. (a) The upper panel is a region of chromosome 17q21 from the UCSC browser showing the genes located in this region. The next panel shows the results from the GABRIEL consortium [9] (each bar represents a SNP and the y-axis shows −log_10_ *P* values for association with asthma). The following three panels show the lung eQTL results for expression levels of *ORMDL3* (blue bars), *GSDMB* (green bars), and *GSDMA* (red bars), respectively. The y-axis represents −log_10_ *P* values derived from the meta-analysis of gene expression. The black horizontal lines are drawn at *P* = 0.05. (b) Boxplots of lung gene expression levels for GSDMA according to genotype groups for SNPs rs3859192 and rs7216389 in 1,111 subjects.

As reported previously [22], [23], eQTLs were often found with the expression of more than one gene underlying a GWAS signal (Table 3). For example, the rs7216389-T allele on chromosome 17q was reported to increase asthma risk with an odds ratio (OR) of 1.45 [10]. This SNP was significantly associated with the expression levels of four genes, *ORMDL3*, *GSDMA*, *GSDMB*, and *CRKRS* (Figure 2a). Although *ORMDL3* was originally suggested [10] to be the gene mediating rs7216389-T's association with asthma, this gene demonstrated the weakest eQTL signal of the four genes in our dataset. Rs7216389-T was positively associated with expression levels of *ORMDL3* (*P* = 1×10^−7^), *CRKRS* (*P* = 1.76×10^−9^) and *GSDMB* (*P* = 4×10^−15^), consistent with the results of Moffat et al. [10] However, this same allele was inversely related to the level of expression of *GSDMA* (*P* = 8.78×10^−25^) (Figure 2b). The strongest eSNP in the 17q asthma susceptibility locus was rs3859192 located in intron 6 of the *GSDMA* gene governing the expression levels of this gene (*P* = 3.55×10^−151^) (Figure 2b).

**Table 3:** Expression eQTL underlying Asthma GWAS hits.

SNP	Gene Symbol	ProbeSet	eQTL *β* (*P* value)			Meta *P* value
			*Laval*	*Groningen*	*UBC*	
rs7216389	Moffatt et al., Nature					
C T*	ORMDL3	100127152_TGI_at	NA	NA	0.30 (9.42E−05)	1.29E−06
	GSDMA	100138145_TGI_at	−0.43 (7.60E−11)	−0.41 (4.38E−08)	−0.42 (8.23E−08)	8.78E−25
	GSDMB	100144966_TGI_at	NA	0.39 (6.42E−08)	NA	4.15E−14
	CRKRS	100152237_TGI_at	−0.35 (4.28E−07)	NA	NA	2.02E−09
	CRKRS	100305614_TGI_at	−0.35 (3.61E−07)	NA	NA	2.79E−09
	CRKRS	100307813_TGI_at	−0.31 (6.95E−06)	NA	NA	1.76E−09
rs2305480	Moffatt et al, NEJM					
A G	ORMDL3	100127152_TGI_at	NA	NA	NA	2.11E−05
	GSDMA	100138145_TGI_at	0.48 (3.18E−13)	0.44 (2.88E−09)	0.50 (7.94E−11)	7.43E−32
	GSDMB	100144966_TGI_at	NA	−0.36 (4.69E−07)	NA	2.52E−12
	CRKRS	100152237_TGI_at	0.29 (2.63E−05)	NA	NA	4.45E−07
	CRKRS	100305614_TGI_at	0.28 (5.27E−05)	NA	NA	3.77E−07
	CRKRS	100307813_TGI_at	NA	NA	NA	4.28E−07
rs3859192**	Moffatt et al, NEJM					
C T	GSDMA	100138145_TGI_at	−0.91 (1.96E−68)	−0.75(3.15E−32)	−0.90(1.20E−54)	3.55E−151
	GSDMB	100144966_TGI_at	NA	NA	NA	3.86E−5
rs2244012	Li et al., JACI					
A G	RAD50	100132153_TGI_at	NA	NA	NA	5.62E−06
rs9273349	Moffatt et al., NEJM					
C T	HLA-DRB	100125275_TGI_at	NA	NA	0.37 (1.17E−06)	2.60E−07
	AGPAT1	100126665_TGI_at	NA	0.47 (5.82E−12)	0.53 (7.23E−13)	1.06E−24
	AGPAT1	100135327_TGI_at	NA	0.36 (3.76E−07)	0.43 (1.05E−08)	7.77E−15
	NA	100160430_TGI_at	NA	−0.48 (3.48E−12)	−0.51 (8.68E−12)	6.78E−24
	HLA-DQB	100300398_TGI_at	NA	0.57 (8.39E−17)	0.55 (1.80E−13)	2.70E−31
	HLA-DRB5	100302940_TGI_at	NA	0.51 (3.48E−13)	0.50 (1.23E−11)	6.28E−25
	HLA-DQB	100302941_TGI_at	NA	0.58 (4.51E−17)	0.55 (1.76E−13)	1.21E−31
	HLA-DQB	100304009_TGI_at	NA	0.58 (3.76E−17)	0.55 (1.23E−13)	6.58E−32
	NA	100311684_TGI_at	NA	0.37 (1.32E−07)	0.35 (4.43E−06)	1.05E−12
	HLA-DQB	100311704_TGI_at	NA	0.62 (1.12E−19)	0.72 (9.13E−24)	2.58E−47
	HLA-DRB	100312946_TGI_at	NA	0.34 (1.43E−06)	0.49 (6.20E−11)	2.22E−16
	HLA-DQA2	100313766_TGI_at	NA	−0.60 (1.93E−18)	−0.61 (1.43E−16)	6.52E−37
rs3894194	Moffatt et al., NEJM					
G A	GSDMA	100138145_TGI_at	−0.75 (1.45E−29)	−0.65 (5.25E−19)	−0.67 (5.83E−19)	2.76E−73
	GSDMB	100144966_TGI_at	NA	NA	NA	1.57E−07
	CRKRS	100307813_TGI_at	NA	NA	−0.32 (5.04E−05)	1.51E−05
rs1295686	Moffatt et al., NEJM					
C T	SLC22A5	100123073_TGI_at	NA	NA	NA	3.49E−05
rs13431828***	Moffatt et al., NEJM					
	IL1RL1	100148162_TGI_at	−0.44 (1.96E−5)	NA	NA	7.48E−08
rs2073643	Moffatt et al., NEJM					
T C	SLC22A5	100123073_TGI_at	0.44 (1.12E−10)	0.31 (7.13E−06)	0.41 (1.69E−08)	9.17E−23
	RAD50	100132153_TGI_at	0.32 (2.72E−06)	NA	NA	4.44E−11
	CDC42SE2	100308155_TGI_at	NA	NA	NA	1.04E−05
rs2786098	Sleiman et al., NEJM					
T G	CRB1	100144551_TGI_at	−0.35 (7.03E−05)	NA	NA	1.08E−08

* Beneath the SNP ID in column 1, the reference allele and risk allele are shown (e.g. for rs7216389, C is the reference allele and T the risk allele).

** Moffatt et al. [9] reported that rs3859192 in the 17q region was associated with asthma (*P* = 1.12×10^−12^). SNP rs3859192 was the most significant eSNP at this locus – strongly associated with GSDMA levels (*P* = 3.55×10^−151^) but weakly associated with GSDMB levels (*P* = 3.86×10^−5^).

*** Moffatt et al. [9] reported that SNPs at the IL1RL1 locus were associated with asthma and the GWAS peak was located at rs3771166. The latter SNP was not a strong lung eSNP (failed to pass 10% FDR). Another SNP at the IL1RL1 locus, rs13431828, was strongly associated with asthma (Moffatt et al. [9], *P* = 1×10^−10^) and IL1RL1 expression level (*P* = 7.48×10^−8^).

**Figure 3:**
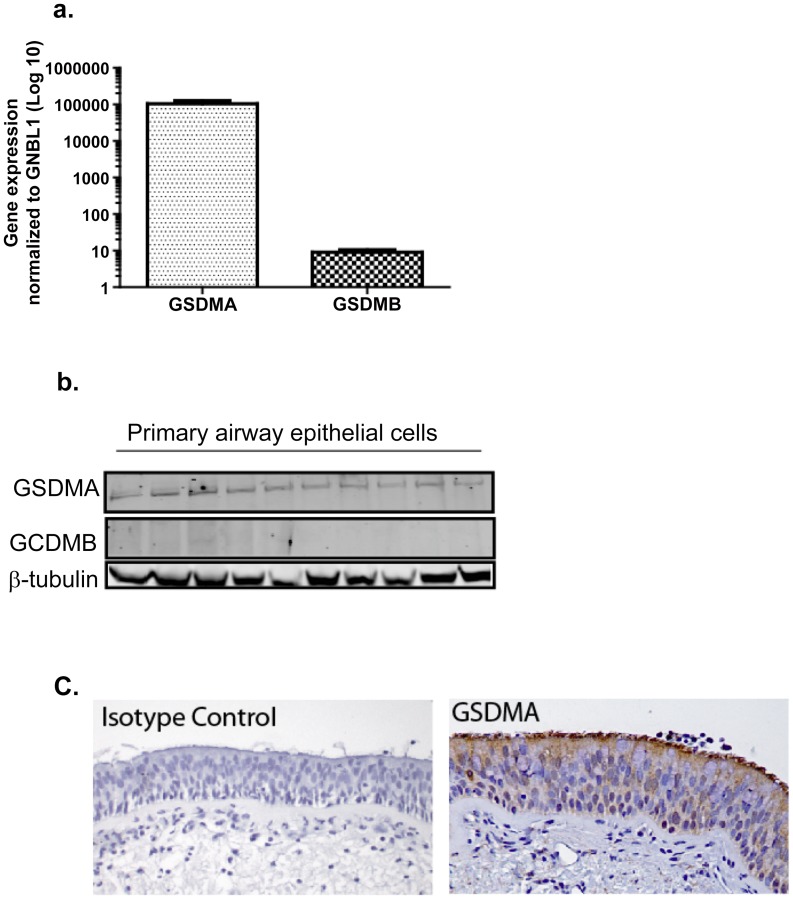
Relative expression of *GSDMA* and *GSDMB*. Primary human airway epithelial cells in monolayer culture were analyzed for; (a) the relative abundance of mRNA for *GSDMA* and *GSDMB* (n = 7) and (b) Protein expression of *GSDMA* and *GSDMB* by Western blot normalized to expression of β-tubulin (n = 10). (c) Representative image of *GSDMA* expression in human conducting airway by immunohistochemistry, which shows expression of *GSDMA* in both basal and apical cells within the airway epithelium.

To determine the cellular source and relative expression of *GSDMB* and *GSDMA* in human lung we performed real-time PCR, and Western blots on primary normal airway epithelial cells and immunohistochemistry on formalin-fixed normal lung tissue (see Text S1 for details). Figure 3 shows abundant mRNA and protein expression of *GSDMA* but little *GSDMB* from primary human lung epithelial cells (Figure 3a and 3b respectively). Figure 3c shows that *GSDMA* is expressed in both apical and basal airway epithelial cells in the conducting airways.

**Figure 4:**
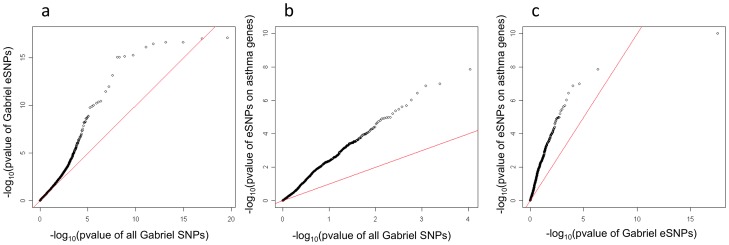
Q–Q plot for the GABRIEL meta-analysis among eSNPs found in the lung eQTL study. Among all SNPs surveyed by the GABRIEL meta-analysis, 60,530 were eSNPs in the Lung eQTL study and considered in this Q-Q plot. As is apparent eQTLs were enriched for lower *P* values (enrichment *P* value<2.2E−16). Panel (a) is GABRIEL eSNPs plotted against GABRIEL SNPs, panel (b) is eSNPs of canonical asthma genes plotted against GABRIEL SNPs and panel (c) is eSNPs of canonical asthma genes plotted against GABRIEL eSNPs.

While most GWAS publications only report the top signals, the GABRIEL study [9] released the asthma association results on all SNPs investigated, allowing an in-depth analysis. For this analysis, we did not limit the list of eSNPs to the most significant SNP for each probeset; all cis eSNPs which passed the 10% FDR for any probe-set were considered. Using this strategy 60,530 of the 567,589 GABRIEL SNPs were eSNPs in lung tissue. These 60,530 eSNPs were enriched for significant association with asthma in the GABRIEL study (Figure 4). This is consistent with previous studies showing that SNPs associated with complex traits are more likely to be eQTLs [24].

### Key Driver Analyses (KDA) of networks

One of the drawbacks of GWAS is the reliance on a large number of statistical tests, which puts the threshold for significance at an extremely low level thereby increasing the chance of missing real associations. Given that asthma is a pulmonary disorder it is reasonable to assume that important molecular drivers are expressed in lung tissue. Therefore, instead of filtering primarily by *P* values to identify loci/genes that explain asthma, we filtered the loci in the GABRIEL [9] dataset by their status as a *cis* acting eSNP. Specifically, all SNPs from the GABRIEL study associated with asthma with *P*<0.01 were translated into genes via our lung eSNP list. A total of 7,613 SNPs were linked to the expression of 739 unique genes.

**Figure 5:**
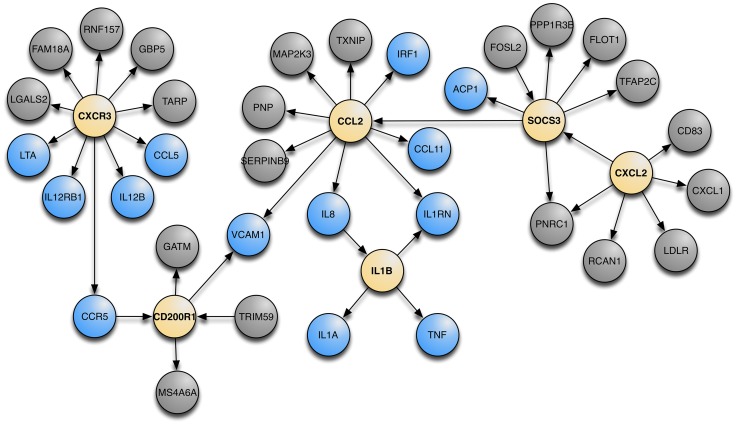
The meta-analysis Bayesian network with the top 6 KDA genes and their nearest neighbors. KDA genes are in yellow, canonical genes are in blue, and all other genes are in grey. *P* values for each of the key driver genes are in Table S3.

Using Bayesian networks constructed on gene expression data we attempted to find the molecular underpinnings of asthma. For this discussion we define a Bayesian network as a probabilistic graphical model that represents a set of random variables (gene expression in this case) and their conditional dependencies (edges) via a directed acyclic graph (see Text S1). The workflow is illustrated in Figure S2. We first identified each gene from the GABRIEL GWAS dataset and collected all genes within 3 edges of that gene on the Bayesian Networks and found the largest coherent sub-network (i.e. the largest network with an observable path between every gene in the network) that contained the highest proportion of GABRIEL genes (Figure S3). We choose to use 3 edges so that we had enough genes to lead to a reasonable biological annotation of the genes in the network, i.e. limiting to 2 edges is too restrictive and results in a small number of relevant genes; on the other hand, using 4 edges leads to broad gene sets and makes it hard to pinpoint pathways. From this sub-network, we performed Key Driver Analyses (KDA) to capture key regulators of asthma [25], [26]. KDA is a method that captures the “hub nodes” conditioning on the direction that one gene influences over other genes and isolating those genes with the strongest influence over the entire network. KDA determines the regulatory components in a directed network for a particular set of genes (i.e. the genes whose expression levels were control by GABRIEL GWAS SNPs). With this analysis, we identified well documented asthma candidate genes [27] that drive genes discovered in GABRIEL. These genes are denoted as canonical asthma genes. Figure 5 shows the top 6 key driver genes (yellow nodes) that control the asthma canonical genes (blue nodes) as well as other genes that are one edge away from the key driver genes (grey nodes). The network when annotated with GeneGo intuitively is described as “Immune Response” (the number of overlap genes is 25, from a 1,346 genes set, with the background being 16,606, *P* = 3.53×10^−20^). The KDA data in Table S3 shows the six genes that remain significant after adjustments for multiple testing.

### Network biology

Networks provide a means for data integration. Above we used the networks to define a central node of the network that drives asthma by integrating lung Bayesian networks, asthma GWAS and literature (canonical genes) to arrive at set of genes that drive the asthma genes in the lung Bayesian network. Similarly we can use the Bayesian networks to rank competing genetic hypotheses around the molecular basis of asthma. It has been shown that the neighborhood milieu of proteins in a network can predict the probable function of a protein for which no function is known [28]. Following the same logic we assessed the sub-network surrounding each candidate causal gene for asthma. The gene with the highest connection to other asthma genes is most likely a causal asthma gene. Chromosomes 2q12 and 17q21 both have strong GWAS hits for asthma. However, these loci harbor a number of genes that could potentially explain these hits^10,11^. Following the workflow shown in Figure S4 [27], we drew inference on causal genes underlying asthma GWAS signals on these two chromosomes (Table 4). Clearly *IL1RL1*'s subnetwork is enriched for canonical asthma genes (*P* = 1.8×10^−07^) making it the most likely gene driving the asthma association on 2q12. The result for 17q21 is less clear, but still points away from *ORMDL3*, the gene first suggested as causal by Moffatt et al. [10]. The sub-network around *GSDMA* is significantly enriched for canonical genes (*P* = 0.005) suggesting that *GSDMA* is the most likely driver behind the asthma association on 17q21.

**Table 4:** Driver genes behind the asthma association on 17q21 and 2q12.

Chr	Genes	*x*	*k*	*P* = value
17	***GSDMA***	**7**	**130**	**0.0049**
	*GSDMB*	4	94	0.054
	*ORMDL3*	3	204	0.2208
2	*IL18R1*	16	381	0.0004
	*IL18RAP*	0	0	1
	***IL1RL1***	**19**	**283**	**1.88×10^−07^**

m = Canonical list [27] (119 genes).

x = Canonical genes within the set of genes within 3 edges of QTL gene.

n = Bayesian – Canonical list (7000 genes).

k = Genes within 3 edges of QTL gene.

## Discussion

One of the primary goals of genetic studies is to identify genes and pathways which contribute to susceptibility to human diseases and towards this goal many GWA studies have discovered SNPs in novel genes [1]–[16]. However, the mechanism by which these SNPs lead to disease susceptibility cannot be directly inferred from GWAS. The discovery of eQTLs has been shown to be a powerful tool in addressing this gap [18]–[20]. Although eQTL analysis of readily available tissue types such as peripheral blood leukocytes and lymphoblastoid cell lines has contributed to the understanding of how genes modulate risk of respiratory diseases, the discovery of lung-specific eQTL is probably more revealing in understanding the pathogenesis of respiratory disease. In this paper, we determined genetic variation and interrogated gene expression in a large collection (n = 1,111) of human lung samples to systematically characterize the genetic architecture of gene expression in this tissue. Owing to the large sample size, we discovered over 17,000 eQTLs. These eQTLs are gene variants that directly or indirectly govern gene expression in the lung and the data set represents a unique resource which we have made available for the use of lung researchers.

To illustrate the power of the resource we merged these data with SNPs known to be strongly associated with asthma and were able to identify the most likely causal gene variants. Chromosome 17q21 is the most consistent locus associated with asthma [9], [10], [29]–[36]. In the original GWAS study [10], the SNPs associated with asthma in the 17q21 susceptibility locus were also associated with transcript levels of *ORMDL3* in lymphoblastoid cell lines, suggesting that *ORDML3* was the causal gene. However, more refined analysis of the same samples, exploiting data generated by the 1000 genomes project, suggested that *GSDMB*, in close proximity to *ORMDL3*, could be the causal gene [9]. Another eQTL study performed in white blood cell RNA samples suggested that many SNPs in the 17q21 regions are associated with transcript levels of both *ORMDL3* and *GSDMB* [32]. Verlaan et al. [37] showed that SNPs in the region demonstrate domain-wide *cis*-regulatory effects suggesting long-range chromatin interactions and they found allele-specific differences in nucleosome distribution and binding of the insulator protein CTCF. A recent study also showed that asthma risk alleles on 17q21 were associated with increased *ORMDL3* and *GSDMA* gene expression and elevated IL-17 secretion in cord blood mononuclear cells [38]. To refine the causal gene/variant, we have interrogated our eQTL results within this locus. Interestingly, the SNPs most strongly associated with the expression of *ORMDL3* are located in the promoter (rs4794820) and intron 1 (rs12603332) of the *ORMDL3* gene, but the P values are not convincing (most significant SNP rs4794820 *P* = 1.0×10^−7^). In addition the SNP rs4794820 is more strongly associated with mRNA expression levels of GSDMB (*P* = 9.3×10^−12^) and GSDMA (*P* = 2.2×10^−46^). Rs4794820 is located between *ORMDL3* and *GSDMA* on 17q21 (see Figure 2a for the location of genes). Considering the transcription orientation of *ORMDL3*, *GSDMA*, and *GSDMB*, rs4794820 lies in the promoter of the three genes and is possibly a pleiotropic regulatory variant or, more likely, in LD with such a regulatory element. We observed a concordance between SNPs associated with transcript levels of *ORMDL3*, *GSDMB* and *GSDMA* (Figure 2a). These genes are likely co-regulated, as proposed previously [32], [37]. However, the most compelling eQTL on 17q21 was observed in the association between the asthma susceptibility SNP (rs3859192 – p = 1.11^−12^ for association with asthma) and the level of expression of *GSDMA* (*P* = 3.55×10^−151^, Figure 2b). In fact, many SNPs on 17q21 were strongly associated with the expression of GSDMA (Figure 2a). Thus our data strongly suggest that the SNPs across this whole locus are associated with asthma because they modulate GSDMA expression in the lung, clearly showing the value of our lung eQTL dataset to refine previous GWAS hits for asthma. It should be noted that correlations between GWAS and eQTL results must be interpreted with caution. Here we related eQTLs in the lung to GWAS studies for asthma which is an airway disease but which is known to involve other tissue types including immune cells. The genetic control of gene expression is tissue specific. Regulatory variants for *GSDMA* identified in the lung may regulate a different gene(s) in another relevant tissue or cell-type for asthma. More functional work in multiple tissues and cell-types measuring gene expression with or without various stimuli will be required to confirm the causal asthma gene on 17q21.

The case for *GSDMA* as the susceptibility gene underlying the 17q GWAS signal is supported by biologic plausibility. GSDMA is a member of the gasdermin family of proteins first identified in the mouse [39]. They have been reported as being expressed in the upper gastrointestinal tract and skin where they are involved in the regulation of apoptosis and act as tumor suppressors [40]. Further support for *GSDMA* as an asthma susceptibility gene is our observation that *GSDMA* is robustly expressed in human airway epithelium (Figure 3). In addition its network neighborhood is enriched in genes involved in immune responses. We examined the genes around *GSDMA* using Bayesian and co-expression networks, and performed functional annotation of these genes (Table S4a–S4b). The GSDMA network neighborhood is enriched for a number of immune response pathways, which are highly relevant to asthma.

By integrating asthma GWAS results with our eSNP and Bayesian network, we were able to identify a network of 34 genes that highlights the molecular underpinnings of asthma (Figure 5 and Table S3). This network of genes is annotated as *inflammatory response* by the online tool DAVID [41] with an enrichment score of 3.27 (*P* = 7×10^−6^). Of particular interest is one Key Driver node, *SOCS3* (suppressor of cytokine signaling 3). *SOCS3* belongs to the *SOCS* family of genes that are cytokine-inducible negative regulators of cytokine signaling and play an important role in T_H_2-mediated allergic responses through control of the balance between T_H_1 and T_H_2 cells. It is implicated in both asthma and atopic dermatitis, as well as in regulating serum IgE levels [42]. Moriwaki and colleagues found that down-regulation of *Socs3* in ovalbumin sensitized mice caused attenuation of eosinophilia and airway hyperresponsiveness generated by ovalbumin challenge [43].

Important discoveries in the field of asthma were made using eQTL mapping in lymphoblastoid cell lines [10]. In this study, we used lung tissue to map lung eQTLs, which is likely the most relevant tissue to study the genetics of lung diseases like asthma. Ideally one would want to relate SNPs to gene expression and to disease phenotype using tissue or cells from individuals affected by the disease of interest [44]. In this study, only a small percentage of the subjects had self-reported asthma. It should be noted that eQTL mapping in liver tissues was successful to identify new susceptibility genes for type 1 diabetes, coronary artery disease and blood lipid levels [19], [20], [45]. These discoveries were made regardless of the medical condition of patients from whom the liver tissues were explanted. Similarly, lung tissue from any source (normal or diseased) is a valid approach to identify the genetic variants that influence gene expression irrespective of disease status. The heterogeneous nature of the tissue we profiled with varying environmental exposures (e.g. smoking) and disease status (e.g. lung cancer) would not be expected to generate associations between SNPs and gene expression but would rather tend to introduce noise into these relationships.

In summary, the present study reports on a comprehensive set of lung eQTLs that complement previous large scale studies of eQTLs in other tissue types [18], [19] and which can be used to shed light on GWAS findings in lung diseases. Using the results of the largest asthma GWA study as an example we show how the lung tissue eQTL dataset can be used to identify the most likely causal genes and pathways. This dataset constitutes an invaluable tool to provide new insights into the pathogenesis of other lung diseases such as chronic obstructive pulmonary disease, lung cancer and cystic fibrosis.

## Materials and Methods

### Ethics statement

All lung tissue samples were obtained in accordance with Institutional Review Board guidelines at the three sites: Laval University (Quebec, Canada), University of British-Columbia (Vancouver, Canada) and Groningen University (Groningen, The Netherlands). All patients provided written informed consent and the study was approved by the ethics committees of the Institut universitaire de cardiologie et de pneumologie de Québec and the UBC-Providence Health Care Research Institute Ethics Board for Laval and UBC, respectively. The study protocol was consistent with the Research Code of the University Medical Center Groningen and Dutch national ethical and professional guidelines (“Code of conduct; Dutch federation of biomedical scientific societies”; http://www.federa.org).

### Lung tissues collection

Lung tissue was collected from patients who underwent lung resectional surgery at the three participating sites; Laval University, University of British Columbia, and University of Groningen. Lung specimens from the Laval site were taken from the Respiratory Health Network Tissue Bank of the FRQS (www.rsr.chus.qc.ca). Subjects' enrollment and lung tissues processing at the three sites are described in Text S1.

### Genome-wide gene expression and genotyping

Gene expression profiles were obtained using a custom Affymetrix array (see GEO platform GPL10379) testing 51,627 non-control probesets. DNA samples were genotyped on the Illumina Human1M-Duo BeadChip array. 409, 363, and 339 patients had both genotyping and gene expression data that passed standard quality controls (see Text S1) in Laval, Groningen, and UBC, respectively. These final datasets (total n = 1,111) were used to discover eQTLs and to identify eSNPs.

### Statistical analysis

The normalized expression data were adjusted for age, gender and smoking status in a robust linear model to accommodate potential outliers in expression level (Figure 1). In parallel, we performed genotype QC to exclude SNPs of low call rate (<0.9) and deviating from Hardy-Weinberg equilibrium (*P*<1×10^−6^). Genotype imputations were based on the cleaned sets. In the end, assayed and imputed genotypes were used to identify *cis* and *trans* acting expression quantitative trait loci (eQTLs) following a method similar to that previously described in Schadt et al. [19]. The strategy for assembling of the asthma candidate gene list (Table S5) and for constructing Bayesian networks, and co-expression modules are given in Text S1.

## Supporting Information

Figure S1 Distribution of the R^2^ values for the relationships between eSNPs and gene expression using a 10% false discovery rate for *cis* acting eQTLs (upper panel) and *trans* acting eQTLs (lower panel).
(PDF)

Figure S2 Workflow to identify key drivers of the lung Bayesian networks. We filtered the asthma GWAS SNPs down only to those SNPs that associate with the trait asthma and have a significant corresponding gene expression pattern in the lung (lung eSNP). The eSNP filtered genes where used to identify the asthma subnetwork from the larger Bayesian network from which KDA was performed.
(PDF)

Figure S3 The largest lung expression subnetwork that has the highest proportion of GABRIEL genes.
(PDF)

Figure S4 Workflow to identify the most likely gene that drives the GWAS asthma association on chromosomes 2 and 17. The logic is that a gene that is surrounded by known asthma genes is more likely to be an asthma gene itself.
(PDF)

Table S1 Mean (Median) of regression r2 in expressional QTLs.
(PDF)

Table S2 (a) cis-eQTLs at 10% FDR. (b) Trans-eQTLs at 10% FDR.
(XLSX)

Table S3 Six Key Driver Analyses (KDA) genes that remains significant after correction for multiple testing.
(PDF)

Table S4 Biological functions of the Bayesian network surrounding *GSDMA*.
(PDF)

Table S5 Asthma candidate genes.
(PDF)

Text S1 Supporting Information.
(DOCX)
