# Correction: Lung eQTLs to Help Reveal the Molecular Underpinnings of Asthma

**DOI:** 10.1371/annotation/80d53ac6-4f5d-4c34-b92b-3fec00d514ac

**Published:** 2012-12-12

**Authors:** Ke Hao, Yohan Bossé, David C. Nickle, Peter D. Paré, Dirkje S. Postma, Michel Laviolette, Andrew Sandford, Tillie L. Hackett, Denise Daley, James C. Hogg, W. Mark Elliott, Christian Couture, Maxime Lamontagne, Corry-Anke Brandsma, Maarten van den Berge, Gerard Koppelman, Alise S. Reicin, Donald W. Nicholson, Vladislav Malkov, Jonathan M. Derry, Christine Suver, Jeffrey A. Tsou, Amit Kulkarni, Chunsheng Zhang, Rupert Vessey, Greg J. Opiteck, Sean P. Curtis, Wim Timens, Don D. Sin

The following information was missing from the Funding section: Y Bossé is a research scholar from the Heart and Stroke Foundation of Canada. DS Postma is the holder of a KNAW (Royal Academy of Arts and Sciences) chair for research. DD Sin holds a Canadian Research Chair in COPD. A Sandford is the recipient of a Canada Research Chair in genetics and a Michael Smith Foundation for Health Research Senior Scholar Award. D Daley is the recipient of a Canada Research Chair in genetic epidemiology and a Michael Smith Foundation for Health Research Scholar Award. PD Paré is a Michael Smith Foundation Distinguished Scholar and the Jacob Churg Distinguished Researcher. This study was funded by Merck Research Laboratories. Support to conduct this study at the Laval site was also supported by the Chaire de pneumologie de la Fondation JD Bégin de l’Université Laval, the Fondation de l’Institut universitaire de cardiologie et de pneumologie de Québec, the Respiratory Health Network of the FRSQ, and the Cancer Research Society. The funders had no role in study design, data collection and analysis, decision to publish, or preparation of the manuscript.

