# Supplementary figures and images for: Lung eQTLs to Help Reveal the Molecular Underpinnings of Asthma
Source: PLoS Genet. 2012 Nov 29;8(11):e1003029. doi: 10.1371/journal.pgen.1003029 (PMC3510026; doi:10.1371/journal.pgen.1003029)

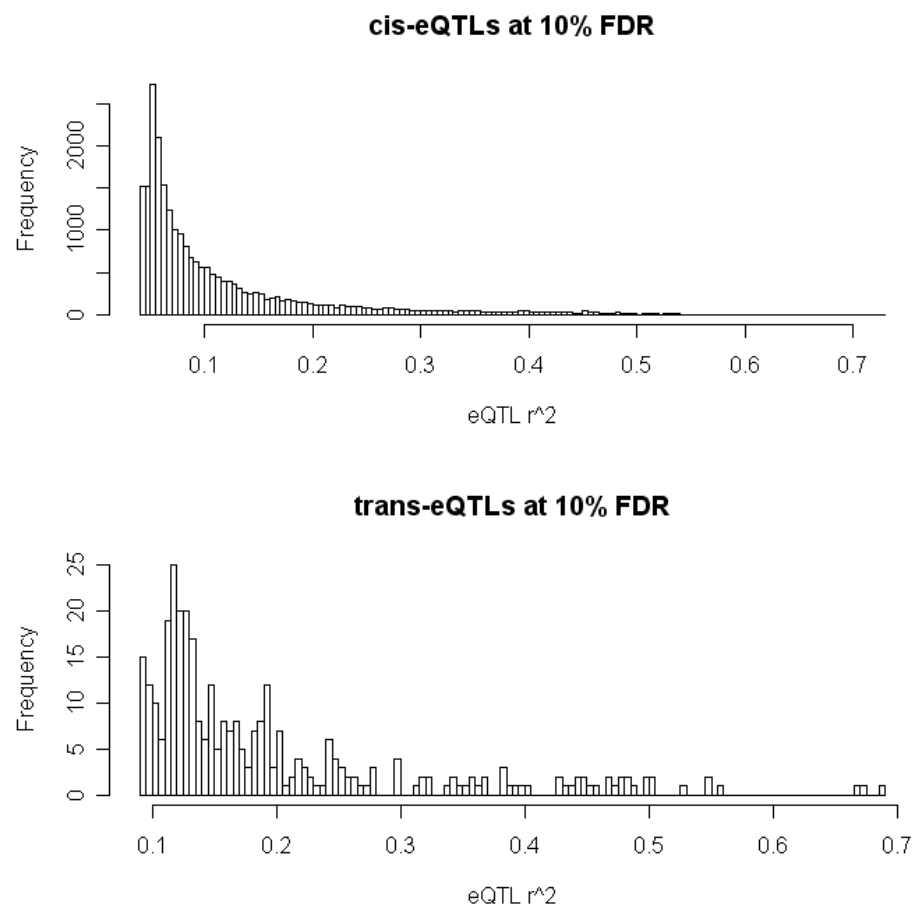

**Figure S1.**

Supplement: Figure S1 — Distribution of the R2 values for the relationships between eSNPs and gene expression using a 10% false discovery rate for cis acting eQTLs (upper panel) and trans acting eQTLs (lower panel). (PDF) [file pgen.1003029.s001.pdf]

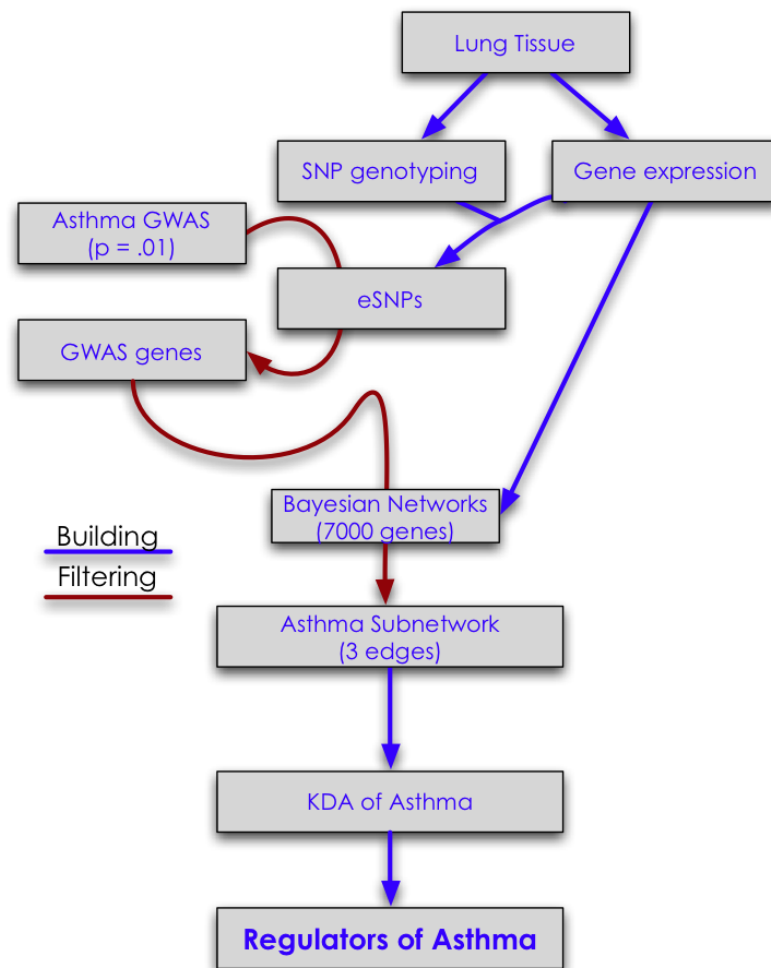

**Figure S2.**

Supplement: Figure S2 — Workflow to identify key drivers of the lung Bayesian networks. We filtered the asthma GWAS SNPs down only to those SNPs that associate with the trait asthma and have a significant corresponding gene expression pattern in the lung (lung eSNP). The eSNP filtered genes where used to identify the asthma subnetwork from the larger Bayesian network from which KDA was performed. (PDF) [file pgen.1003029.s002.pdf]

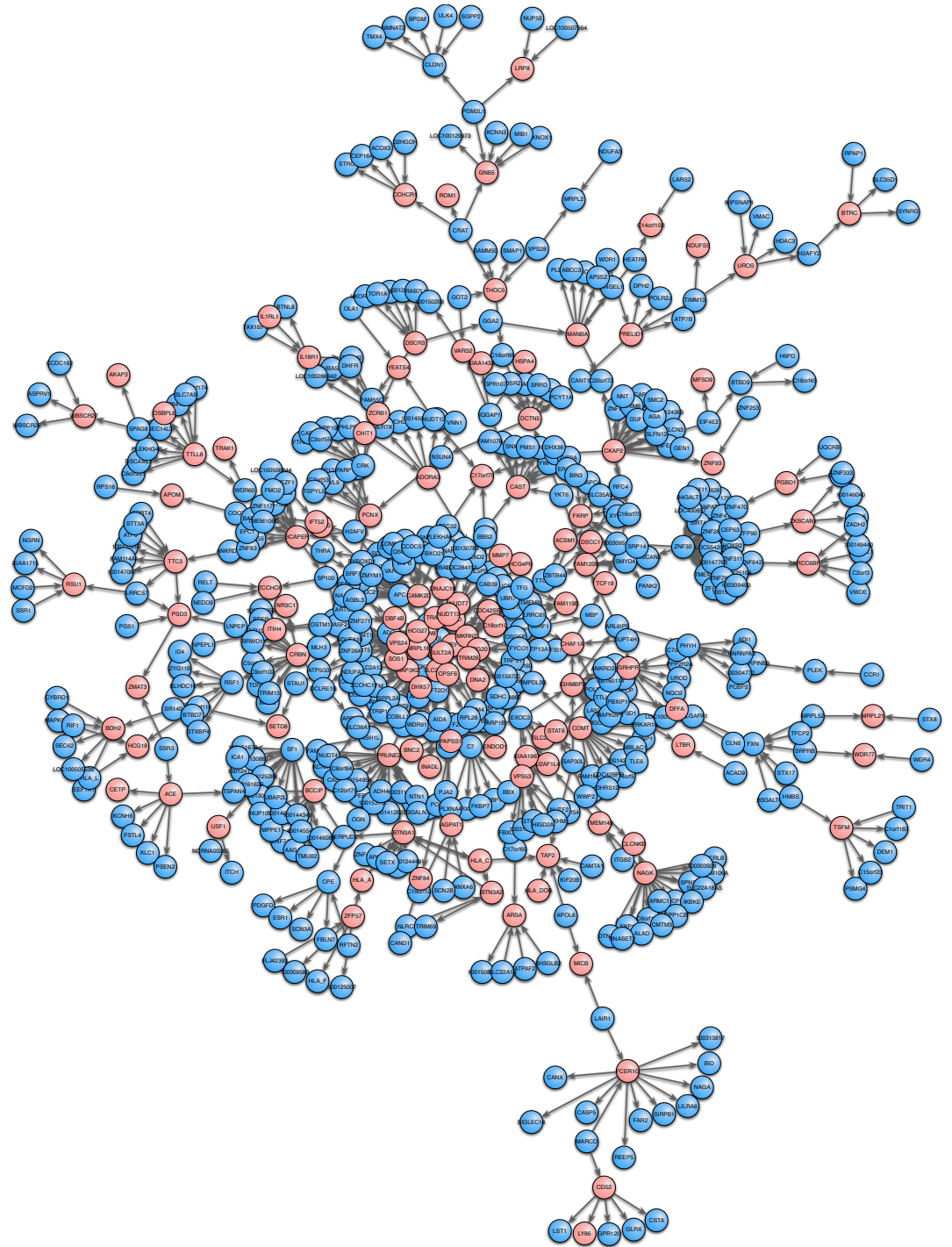

**Figure S3.**

Supplement: Figure S3 — The largest lung expression subnetwork that has the highest proportion of GABRIEL genes. (PDF) [file pgen.1003029.s003.pdf]

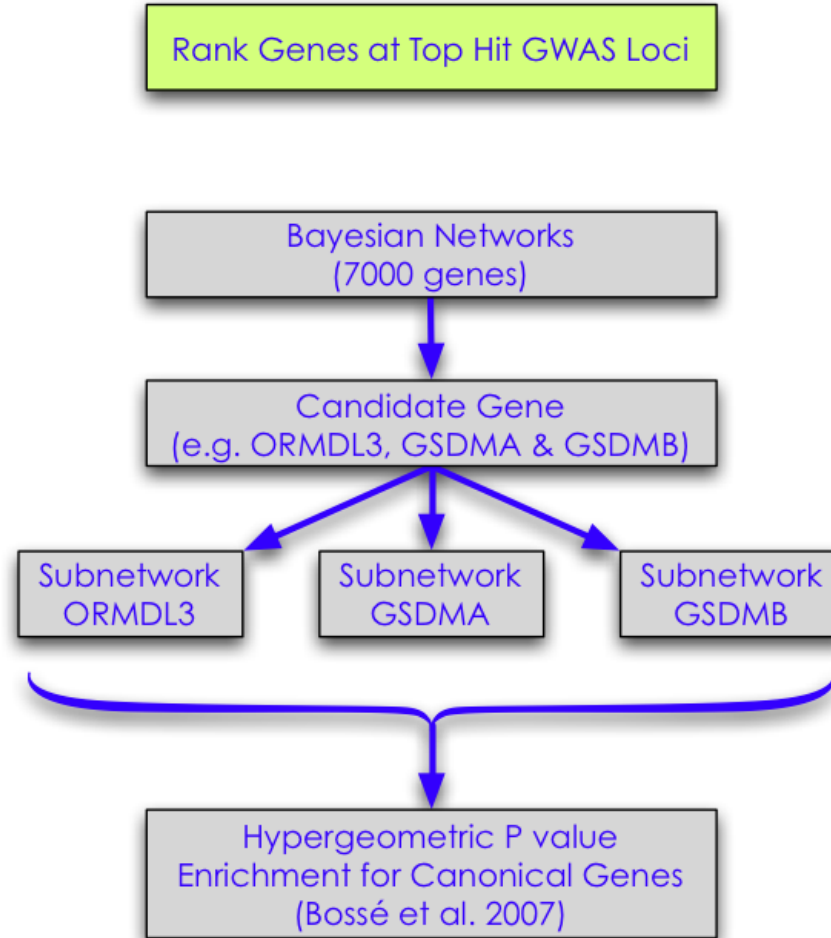

**Figure S4.**

Supplement: Figure S4 — Workflow to identify the most likely gene that drives the GWAS asthma association on chromosomes 2 and 17. The logic is that a gene that is surrounded by known asthma genes is more likely to be an asthma gene itself. (PDF) [file pgen.1003029.s004.pdf]
