# Supplementary material for: Lung eQTLs to Help Reveal the Molecular Underpinnings of Asthma
Source: PLoS Genet. 2012 Nov 29;8(11):e1003029. doi: 10.1371/journal.pgen.1003029 (PMC3510026; doi:10.1371/journal.pgen.1003029)
Supplement: Table S1 — Mean (Median) of regression r2 in expressional QTLs. (PDF) [file pgen.1003029.s005.pdf]

Table S1. Mean (Median) of regression r^2 in expressional QTLs

**Cohort**		1% FDR	5% FDR	10% FDR
Laval	*cis*-eQTLs	0.140(0.098)	0.122(0.080)	0.112(0.071)
	trans-eQTLs	0.203(0.145)	0.194(0.135)	0.181(0.128)
Groningen	*cis*-eQTLs	0.143(0.106)	0.124(0.087)	0.112(0.076)
	trans-eQTLs	0.228(0.192)	0.205(0.165)	0.191(0.149)
UBC	*cis*-eQTLs	0.157(0.113)	0.138(0.096)	0.128(0.086)
	trans-eQTLs	0.241(0.188)	0.224(0.182)	0.218(0.178)
