# Supplementary material for: Lung eQTLs to Help Reveal the Molecular Underpinnings of Asthma
Source: PLoS Genet. 2012 Nov 29;8(11):e1003029. doi: 10.1371/journal.pgen.1003029 (PMC3510026; doi:10.1371/journal.pgen.1003029)
Supplement: Table S3 — Six Key Driver Analyses (KDA) genes that remains significant after correction for multiple testing. (PDF) [file pgen.1003029.s007.pdf]

Table S3. Six Key Driver Analyses (KDA) genes that remains significant after correction for multiple testing

**Key Driver**	***P***	**Corrected-*P***	**q-value**	**Hits***
CXCR3	3.27E-10	1.17E-06	3.28E-04	16
CCL2	8.71E-07	3.10E-03	3.28E-04	13
CXCL2	3.00E-06	1.07E-02	3.28E-04	10
SOCS3	1.95E-05	6.93E-02	1.72E-03	12
IL1B	6.78E-05	2.42E-01	4.13E-03	6
CD200R1	9.98E-05	3.56E-01	4.59E-03	5

* Hits is the number of gene that are from the canonical list that are also three edges away from the Key Driver gene
