# Supplementary material for: Lung eQTLs to Help Reveal the Molecular Underpinnings of Asthma
Source: PLoS Genet. 2012 Nov 29;8(11):e1003029. doi: 10.1371/journal.pgen.1003029 (PMC3510026; doi:10.1371/journal.pgen.1003029)
Supplement: Table S4 — Biological functions of the Bayesian network surrounding GSDMA. (PDF) [file pgen.1003029.s008.pdf]

Table S4a. Biological functions of the Bayesian network surrounding *GSDMA*

**Name**	**Expectation***	**Source**	***P***
immune response-regulating cell surface receptor signaling pathway	0.002479	GO_process	8.34E-07
T cell receptor signaling pathway	0.006371	GO_process	2.15E-06
leukocyte proliferation	0.009159	GO_process	3.09E-06
immune response-regulating signaling pathway	0.011614	GO_process	3.92E-06
antigen receptor-mediated signaling pathway	0.02516	GO_process	8.56E-06
immune response-activating cell surface receptor signaling pathway	0.031045	GO_process	1.06E-05
regulation of immune response	0.032261	GO_process	1.10E-05
lymphocyte proliferation	0.035811	GO_process	1.22E-05
mononuclear cell proliferation	0.038009	GO_process	1.30E-05

*Corrected for multiple testing.

Table S4b. Biological functions of the co-expression module harboring *GS DMA*

name	Expectation*	source	*P*
Allograft rejection	1.01E-08	kegg	3.68E-12
antigen processing and presentation	6.64E-08	GO_process	2.43E-11
immune system process	4.30E-07	GO_process	1.58E-10
Graft-versus-host disease	1.41E-06	kegg	5.15E-10
Autoimmune thyroid disease	1.76E-06	kegg	6.46E-10
Viral myocarditis	2.18E-06	kegg	8.00E-10
immune response	3.33E-06	GO_process	1.22E-09
Type I diabetes mellitus	1.55E-05	kegg	5.68E-09
antigen processing and presentation of peptide antigen	6.09E-05	GO_process	2.23E-08
Antigen processing and presentation	1.29E-04	kegg	4.72E-08
Intestinal immune network for IgA production	1.81E-04	kegg	6.62E-08
Cell adhesion molecules (CAMs)	2.21E-04	kegg	8.08E-08
regulation of immune system process	0.001243	GO_process	4.55E-07
Asthma	0.002561	kegg	9.39E-07
response to virus	0.016771	GO_process	6.19E-06
T cell activation	0.020734	GO_process	7.67E-06
multi-organism process	0.06281	GO_process	2.38E-05
regulation of lymphocyte activation	0.077555	GO_process	2.96E-05
lymphocyte activation	0.081983	GO_process	3.13E-05

*Corrected for multiple testing.
