# Supplementary material for: Lung eQTLs to Help Reveal the Molecular Underpinnings of Asthma
Source: PLoS Genet. 2012 Nov 29;8(11):e1003029. doi: 10.1371/journal.pgen.1003029 (PMC3510026; doi:10.1371/journal.pgen.1003029)
Supplement: Table S5 — Asthma candidate genes. (PDF) [file pgen.1003029.s009.pdf]

Asthma candidate genes
ABO
ACE
ACP1
ACVR1
ADA
ADAM33
ADAM8
ADCY9
ADH5
AGT
AICDA
AKR1C3
ALDH2
ALOX15
ALOX5AP
ANXA2
AOAH
ARG1
ARG2
BAT1
BCL2
BCL2L1
BDKRB1
BDKRB2
BDNF
BMP2
BMP4
BMP7
BMPR1A
BMPR1B
C3
C3AR1
C5
C5orf20
CAMP
CAT
CCL11
CCL2
CCL28
CCR1
CCR3

CCR5
CCR9
CD14
CD28
CD40
CD69
CD86
CDH1
CDKN1A
CFL1
CFTR
CHI3L1
CHIA
CHIT1
CHML
CHRM1
CHRM2
CHRM3
CLCA1
CMA1
COL1A1
COMT
CREB1
CRHR1
CRHR2
CRP
CSF2RB
CTLA4
CTNNA1
CTNNA3
CX3CR1
CXCL10
CYBA
CYP1A1
CYP1A2
CYP2C9
CYP2J2
CYP2R1
CYP4F3
CYSLTR1
CYSLTR2
DAP3

DEFB1
DPP10
EDNRA
EDNRB
EFNA1
EGFR
EHF
ELAC2
ELF5
EPHX2
F2RL1
FCAR
FCER1G
FCGR2A
FGF2
FGFBP2
FLG
FLT3LG
FMR1
FOXP3
FUT2
FUT3
FYN
GATA3
GC
GCLC
GHRL
GIF
GNAS
GNB1
GPR44
GPX1
GSTA1
GSTM3
GSTO2
GSTP1
GSTT1
HAVCR1
HAVCR2
HEXB
HLA-A
HLA-B

HLA-C
HLA-DRB3
HLA-G
HNMT
HRH2
IFNA1
IFNAR1
IFNGR1
IFNGR2
IKBKAP
IL10
IL10RA
IL12A
IL12RB1
IL12RB2
IL13RA1
IL15
IL16
IL17F
IL18
IL18R1
IL18RAP
IL19
IL1A
IL1R1
IL1RL1
IL1RN
IL21
IL27
IL31
IL8
IL9R
INHA
INPP4A
ITGA4
ITGA6
ITGB3
ITGB7
ITK
ITLN1
JUND
KCNMB1

KCNS3
KDR
LAMA5
LELP1
LEP
LEPR
LGALS3
LTA4H
LTB
LTB4R
LTC4S
MBL2
MICB
MIF
MMP1
MMP21
MMP28
MPO
MS4A1
MS4A3
MTHFR
MUC5AC
MUC7
MYH11
MYLK
NAT1
NAT2
NFKB2
NFKBIL1
NOD2
NOS1
NOS3
NOX4
NPSR1
NQO1
NR3C1
NRIP2
NTF3
ORMDL3
PAFAH1B1
PDGFA
PDGFRA

PHF11
PLA2G4A
PLA2G7
PLAUR
PMCH
PPARG
PTAFR
PTGDR
PTGER1
PTGER2
PTGER3
PTGER4
PTGFR
PTGIR
PTGS1
PTGS2
PTPRD
RASGRP4
RIPK2
RNASE3
ROM1
RUNX1
SCGB1A1
SCGB3A2
SELP
SERBP1
SERPINA3
SERPINB3
SFRP1
SIGIRR
SIGLEC8
SLC11A1
SLC26A4
SMAD2
SOCS1
SOD2
SPINK5
SPP1
STAT2
STAT3
STAT4
TAC1

TAGLN
TBX21
TBXA2R
TGFB1
TGFB2
TGFBR1
TIMD4
TJP1
TLR1
TLR10
TLR2
TLR3
TLR6
TLR7
TLR8
TLR9
TNC
TNFRSF10A
TNFRSF10B
TNFRSF14
TNXB
TPSD1
TPSG1
TPT1
TSLP
VDR
VEGFA
WDR36
XDH
