# Supplementary material for: Lung eQTLs to Help Reveal the Molecular Underpinnings of Asthma
Source: PLoS Genet. 2012 Nov 29;8(11):e1003029. doi: 10.1371/journal.pgen.1003029 (PMC3510026; doi:10.1371/journal.pgen.1003029)
Supplement: Text S1 — Supporting Information. (DOCX) [file pgen.1003029.s010.docx]

**Supplementary Materials -- Text**

**Title**:

Lung eQTLs to help reveal the molecular underpinnings of asthma

**Authors**:

Ke Hao, Yohan Bossé, David C. Nickle, Peter D. Paré, Dirkje S. Postma, Michel Laviolette, Andrew Sandford, Tillie L. Hackett, Denise Daley, James C. Hogg, W Mark Elliott, Christian Couture, Maxime Lamontagne, Corry-Anke Brandsma, Maarten van den Berge, Gerard Koppelman, Alise S. Reicin, Donald W Nicholson, Vladislav Malkov, Jonathan M. Derry, Christine Suver, Jeffrey A. Tsou, Amit Kulkarni, Chunsheng Zhang, Rupert Vessey, Greg J. Opiteck, Sean P. Curtis, Wim Timens, Don D. Sin

**MATERIALS AND METHODS**

**Lung tissues collection**

The study is based on non-tumor lung tissue samples from 1,424 individuals (500, 487, and 437 subjects from Laval, Groningen, and UBC, respectively) with corresponding clinical data. The lung specimens were collected from patients undergoing lung surgery at three academic sites: Laval University (Quebec, Canada), University of British Columbia (Vancouver, Canada) and University Medical Center Groningen (Groningen, The Netherlands). All lung tissue samples were obtained in accordance with Institutional Review Board guidelines.

**Subject enrollment: Laval site**

All patients provided written informed consent and the study was approved by the ethics committees of the Institut universitaire de cardiologie et de pneumologie de Québec. Lung tissues were obtained from patients undergoing lung cancer surgery between April 2004 and December 2008. The 500 specimens were taken from the Respiratory Health Network Tissue Bank of the Fonds de la Recherche en Santé du Québec. After surgical removal, lung specimens were immediately examined by a pulmonary pathologist. After processing for pathological diagnosis and staging, a non-neoplastic sample of pulmonary parenchyma (2-5 cm^3^) was harvested from a site distant from the tumor. The research specimens were immediately divided into smaller fragments (~0.5 cm^3^) placed in 5 ml cryovials and snap-frozen in liquid nitrogen. The cryovials were then transported in dry ice to a local biobank where they were stored at -80^o^C until further processing. The time from surgical removal to storage was between 15 to 30 minutes. The subjects’ smoking history was documented including current smoking status, number of pack-years, and year of smoking cessation (for former-smokers). Preoperatively, patients underwent pulmonary function testing in which lung volumes, forced expiratory volume in 1 sec (FEV_1_), forced vital capacity (FVC), and diffusion capacity for carbon monoxide (DLco) were determined according to the American Thoracic Society (ATS) guidelines[[1](#_ENREF_1),[2](#_ENREF_2)]. FEV_1_ and FVC values were used to define COPD in accordance with the guidelines of the Global initiative for chronic Obstructive Lung Disease (GOLD)[[3](#_ENREF_3)]. Patients’ medical charts were abstracted for co-morbidities including asthma, cardiac diseases and type II diabetes. Cancer types and stages were taken from the pathology report.

**Subject enrollment: UBC site**

All patients provided written informed consent and the study was approved by the ethics committees of the UBC-Providence Health Care Research Ethics Board. The 437 lung tissue samples from UBC were part of a long standing tissue bank established at St Paul’s Hospital. The majority of the samples were from patients undergoing resection of small peripheral lung lesions (n=369). Prior to surgery the patients had lung function and completed a detailed questionnaire modified from the ATS questionnaire detailing smoking history, medication use and co-morbid conditions. Most subjects had measurements of lung function immediately before surgery. The lung function data used for analysis were that obtained from tests done as closely as possible in time to the acquisition of tissue. Lung function tests included measurements of lung volumes, FEV_1_, FVC, and DLco according to the ATS guidelines[[1](#_ENREF_1),[2](#_ENREF_2)]. FEV_1_ and FVC values were used to define COPD in accordance with the GOLD guidelines[[3](#_ENREF_3)]. Cancer types and stages were taken from the pathology report.

Additional samples were obtained at autopsy (n=39) or at the time of lung transplantation (22 diseased lungs from recipients and 7 donor lungs which for various reasons could not be used for transplantation). Tissues from UBC were collected and processed as described previously[[4](#_ENREF_4)]. Immediately following resection, the lung or lobe was obtained from the operating or autopsy room and after the clinical specimens of the lesion, lymph nodes and the resection margin were obtained the lobes and lungs were inflated using a 50% mixture of Cryomatrix^R^ and saline and frozen in liquid nitrogen fumes. The time between resection and freezing averaged ~ 1 hour. The frozen lungs and lobes were then cut into 7-15 two cm thick slices using a band saw and multiple randomly stratified blocks were acquired (1-3/slice) using a power driven hole saw fitted with a 1.5 cm diameter bit. The frozen “cores” were stored at -80^o^ C for later RNA and DNA extraction.

**Subject enrollment: Groningen site**

The study protocol was consistent with the Research Code of the University Medical Center Groningen and Dutch national ethical and professional guidelines (“Code of conduct; Dutch federation of biomedical scientific societies”; http://www.federa.org). The 487 lung tissue samples from Groningen were part of a long standing tissue bank established at the University Medical Center Groningen. The specimens were obtained at surgery from patients with various lung diseases. A large number of the samples were from patients undergoing therapeutic resection for lung tumors (n=153). Immediately following resection, the lung or lobe was obtained from the operating room and processed for pathological diagnosis and staging. After this procedure a non-neoplastic pulmonary parenchyma sample (2-5 cm^3^) was harvested from a site distant from the tumor. Many lung tissue samples were obtained at the time of lung transplantation (290 diseased lungs from recipients and 24 donor lungs where for various reasons the lungs could not be used for transplantation). The research specimens were immediately divided into smaller fragments (~1 cm^3^), snap-frozen in liquid isopentane, and stored at -80^o^C. The time between resection and freezing averaged ~ 1 hour. Preoperatively, many of the patients underwent pulmonary function testing in which lung volumes, FEV_1_, FVC, and DL_CO_ were determined according to the ATS guidelines[[1](#_ENREF_1),[2](#_ENREF_2)]. FEV_1_ and FVC values were used to define COPD in accordance with the GOLD guidelines[[3](#_ENREF_3)]. Patients’ medical charts were surveyed for co-morbidities including asthma, cardiac disease and diabetes. Smoking history was collected including current smoking status, number of pack-years, and year of smoking cessation (for former-smokers). Cancer types and stages were taken from the pathology report.

**Gene Expression and Genotyping Assays**

Samples were shipped to Rosetta Inpharmatics Gene Expression Laboratory (Seattle, WA) for extraction, amplification, labeling, and microarray processing. Genomic DNA was extracted from whole blood (n=100) and buffy coat (n=400) in the Laval samples. DNA from Groningen and UBC were extracted directly from the lung specimens. Total RNA was extracted from the lung tissues. RNA converted to fluorescently labeled cRNA was hybridized to a custom Affymetrix HU133 array, consists of 751 control probesets and 51,627 non-control probesets, predominantly from REFSEQ, GenBank, dbEST and ENSEMBL. All probes were free of known polymorphisms. A number of samples failed Rosetta’s quality controls that are routinely checked throughout the Affymetrix protocol, leaving 479, 445 and 405 subjects with gene expression data from Laval, Groningen and UBC, respectively. In parallel, DNA sample was genotyped on the Illumina Human1M-Duo BeadChip array, and 426, 421 and 390 samples were successfully genotyped for Laval, Groningen and UBC, respectively.

**Gene Expression QC, Normalization and Adjustment**

Expression values were extracted using the Robust Multichip Average (RMA) method[[5](#_ENREF_5)] as implemented in the Affymetrix Power Tools (APT) software. The quality of the arrays was judged based on multiple quality control parameters which check for standard Affymetrix quality control metrics, image artifacts, and outliers based on log intensity distribution, RMA byproducts and principal component analysis. A complete description of quality control assessment can be found in the supplementary materials (see GeneExpressionQC_Laval_v2.pdf, GeneExpressionQC_GRNG_v2.pdf, and GeneExpressionQC_UBC_v2.pdf). A total of 4 out of 479 expression arrays in the Laval set were excluded based on gene expression quality control filters. For Groningen 16 out of 445 arrays and for UBC 10 out of 405 arrays were removed based on the same quality control filters. The complete dataset has been deposited in the National Center for Biotechnology Information’s Gene Expression Omnibus (GEO, http://www.ncbi.nlm.nih.gov/geo/) repository and is accessible through GEO Series accession number GSE23546 (http://www.ncbi.nlm.nih.gov/geo/query/acc.cgi?token=xbobfamguyoewze&acc=GSE23546).

**Transcripts expressed in lung tissue**

Following quality control filters, gene expression data from 475, 429, and 395 unique samples were available for Laval, Groningen, and UBC, respectively. The "present/absent" calls were made by comparing the perfect match and mis-match probes using Affymetrix MAS 5.0 algorithm. A large fraction of the probesets/transcripts were expressed ("present") in lung: 64.4% of the transcripts were "present" for at least half of the samples; and 69.1% of the transcripts were called "present" for at least 1/3 of the samples.

**Expression trait processing**

The normalized expression data were adjusted for age, gender and smoking status in a robust linear model to accommodate potential outliers in expression level. Further, we applied an inverse normalization technique to the adjusted expression values. Following these adjustments, expression levels for every gene showed standard normal distribution, ready for eQTL mapping with linear regression.

**Genotype Data QC**

Arrays with call rates < 98% were first excluded. Gender was confirmed using PLINK[[6](#_ENREF_6)]. Cryptic relatedness and duplicates were identified by calculating the identity by state (IBS) distance for every subject pair within each cohort. 25 and 6 duplicates in the Groningen and UBC cohorts were identified, respectively. In each duplicate pair, we excluded the individual with the lower call rate. In each duplicate pair, we excluded the individual with the lower call rate. EIGENSTRAT was used to infer ethnicity[[7](#_ENREF_7)], and 3, 9 and 25 non-European subjects were excluded in Laval, Groningen, and UBC, respectively. Furthermore, we conducted SNP-wise quality filtering, based on exclusion criteria of (1) minor allele frequency (MAF) < 1%; (2) SNP call rate < 90% and (3) deviation from Hardy-Weinberg equilibrium (HWE) *P* <1 x 10^-6^. In Laval, 159313, 2747 and 676 SNPs were excluded on the three criteria, respectively. In Groningen, 168501, 2333 and 724 SNPs were excluded, and in UBC, 163328, 2953 and 595 SNPs were excluded. The resulting genotype data were fed into the MACH program[[8](#_ENREF_8)] for genotype imputation using HapMap release 22 template. In total, 2,507,647 autosomal SNPs were imputed with high confidence (r^2^ > 0.3) and had an MAF > 0.01, HWE test *P* > 10 x 10^-6^ and < 5% missing data in all samples. On chromosomes X and Y, we restricted our analysis to assayed SNPs.

**Overlap between gene expression and genotyping QC**

409, 363, and 339 patients had both genotyping and gene expression data that passed all quality controls in Laval, Groningen, and UBC, respectively. These final datasets (total n=1,111) were used to discover eQTLs.

**Expression QTLs**

*Cis* and *trans* acting expression quantitative trait loci (eQTLs) were identified using a method similar to that previously described[[9](#_ENREF_9)]. Briefly, eQTLs quantify the association between transcript level and SNP genotype. We surveyed all transcript-SNP pairs using a linear regression model, T_i_ ~ β_0_ + β_1_G_j_, and conducted Wald's test on H_0_: β_1_=0. A *cis* eQTL was identified if SNP genotype was significantly related to a transcript probeset within 1Mb. All other eQTLs were categorized as *trans*-eQTLs. Further, we randomized the IDs in the gene expression file, destroying gene-SNP associations but keeping the gene-gene correlation structure intact. Calculation of eQTLs was repeated on permuted data. Given the vast number of tests in each eQTL run, we found three permutation iterations yielded a stable null distribution of eQTL *P* values. We quantified the false discovery rate (FDR) for *cis*- and *trans*-eQTLs separately. The characteristics of eQTLs at 1%, 5% and 10% are presented in Table S1, and we used the eQTLs of 10% FDR in the main analysis. We also noticed that the replication rate increased as a function of the statistical stringency (i.e., lower FDR cutoff). For example, 59.6% and 69.8% of Laval cis-eQTLs discovered by using 5% and 1% FDR cutoff levels, respectively, were confirmed in the other two cohorts. 82.3% and 86.4% of Laval *trans*-eQTLs discovered at 5% and 1% FDR levels, respectively, were confirmed in the other two cohorts. After within-cohort analysis, we conducted a meta-analysis on the three cohorts by combining the coefficients (β) and *P* values using a fixed effects model[[10](#_ENREF_10)].

Putative eQTLs could be detected in the presence of SNPs within probe annealing sites[[11](#_ENREF_11),[12](#_ENREF_12)]. For example, such SNPs could influence the hybridization of the probe. If the SNP at the probe site was (or was in LD with) one that we genotyped the effect on probe hybridization could create a false positive eQTL. We employed several strategies to safeguard against this confounder. First, in designing the custom Affymetrix HU133 array, we carefully selected probes to avoid all known SNPs. Second, after the experiments, we masked out probes that harbor newly documented SNPs by customizing the CDF file. Last, the Affymetrix expression array includes 11 separate probes for each probeset and we applied the RMA algorithm for expression level quantification, where outlier probes had little impact.

**Interpretation of public GWAS data on asthma using lung eQTLs**

One of the primary utilities of eQTLs is to assign functional significance to GWAS findings. We constructed a list of asthma GWAS “hits” from the NHGRI public catalog[[13](#_ENREF_13)] using only the most stringent associations according to the strict guidelines on significance levels for replication, and by reviewing the literature[[14-26](#_ENREF_14)]. We have termed the asthma GWAS hits which are listed in the NHGRI catalog as a "high confidence asthma set" (n=25 SNPs). In addition, we relaxed the criteria and included more published asthma GWAS “hits” to form a non-overlapping "broad asthma set" (n=91 SNPs). The high confidence genes, SNPs and traits for the asthma analysis, as well as the direction of their association with asthma and the effect of the susceptibility SNPs on expression are shown in **Table 3**. Among the "high confidence asthma set", 10 SNPs were also eSNPs (40% of the set and hypergeometric p-values=0.0033); 27 SNPs from the "broad asthma set" were eSNPs (30% of the set and hypergeometric p-values=0.0040).

**Correlation between gene expression and clinical phenotypes**

For a given clinical phenotype, *T_j_*, we computed its correlation with the expression level of all genes one by one. In brief, when *T_j_* being a categorical variable, we computed correlation using Kruskal-Wallis test, and when *T_j_* being continuous variable, we applied Spearman's correlation. We surveyed phenotype of age, gender, smoking, preBD FVC and preBD FEV_1_. False discovery rate (FDR) was calculated using the R Q-value package[[27](#_ENREF_27)]. Further, we visualize the correlation *P* values in a histogram. If the *P* value distribution was skew to the left, we conclude *T_j_* is correlated with gene expression.

In total, we found two groups of phenotypes associated with gene expression. (1) Health and disease status, e.g. BMI, COPD and FEV1, and (2) life-style and medication parameters (e.g. smoking or statin use). However, such correlations cannot directly infer causality, and adjusting the expression by these phenotypes may jeopardize eQTL mapping. For sample, if the covariate is on the functional pathway in which eSNPs control lung gene expression, adjusting such covariate could obscure the results. In this paper, we only adjusted expression levels by age, gender and smoking exposure because they have profound impact and are less likely to reside on the genotype-expression causal pathway. In detail, smoking exposure was captured by three variables. (1) Smoking status was coded as a discrete variable of current, former or never smokers; (2) pack-year and (3) the years since smoking cessation. We carefully adjusted for the three smoking exposure variable in eQTL analysis.

**Co-expression modules**

We sorted the transcripts by the *P* values of their strongest eQTL, and selected the top 7000 transcripts for constructing a weighted gene co-expression network for each of the three sites[[28](#_ENREF_28)]. The weighted network analysis begins with a matrix of the Pearson correlations between all gene pairs, then converts the correlation matrix into an adjacency matrix using a power function *f(x)=x^β^*. The parameter *β* of the power function is determined in such a way that the resulting adjacency matrix, i.e., the weighted co-expression network, is approximately scale-free. To measure how well a network satisfies a scale-free topology, we use the fitting index proposed by Zhang & Horvath[[28](#_ENREF_28)], i.e., the model fitting index *R^2^* of the linear model that regresses *log(p(k))* on *log(k)* where *k* is connectivity and *p(k)* is the frequency distribution of connectivity. The fitting index of a perfect scale-free network is 1. For each dataset, we selected the smallest *β* which led to an approximately scale free network with the truncated scale free fitting index *R^2^* greater than 0.8.

To explore the modular structure of the co-expression network, the adjacency matrix was further transformed into a topological overlap matrix[[29](#_ENREF_29)]. The topological overlap between two genes reflects not only their direct interaction but also their indirect interactions through all the other genes in the network. Previous studies[[28](#_ENREF_28),[29](#_ENREF_29)] have shown that topological overlap leads to more cohesive and more biologically meaningful modules. To identify modules of highly co-regulated genes, we used average linkage hierarchical clustering to group genes based on the topological overlap of their connectivity, followed by a dynamic cut-tree algorithm to cut clustering dendrogram branches into gene modules[[30](#_ENREF_30)]. To distinguish between modules, each module was assigned a unique color identifier, with the remaining, poorly connected genes colored grey.

**Bayesian Networks**

Bayesian networks are directed acyclic graphs in which the edges of the graph are defined by conditional probabilities that characterize the distribution of states of each node given the state of its parents[[31](#_ENREF_31)]. The network topology defines a partitioned joint probability distribution over all nodes in a network, such that the probability distribution of states of a node depends only on the states of its parent nodes: formally, a joint probability distribution $p(X)$ on a set of nodes $X$ can be decomposed as $p(X)=\prod_{i} p(X^{i}|\text{Pa}(X^{i}))$, where $\text{Pa}(X^{i})$ represents the parent set of $X^{i}$. In our networks, each node represents a gene. These conditional probabilities reflect not only relationships between genes, but also the stochastic nature of these relationships, as well as noise in the data used to reconstruct the network.

Bayes formula allows us to determine the likelihood of a network model M given observed data D as a function of our prior belief that the model is correct and the probability of the observed data given the model: P(M | D) ~ P(D|M) * P(M). The number of possible network structures grows super-exponentially with the number of nodes, so an exhaustive search of all possible structures to find the one best supported by the data is not feasible, even for a relatively small number of nodes. We employed Monte Carlo Markov Chain[[32](#_ENREF_32)] simulation to identify thousands of different potentially plausible networks, which were then combined to obtain a consensus network (see below). Each reconstruction began with a random network. Small random changes were then made to the network by flipping, adding, or deleting individual edges, ultimately accepting those changes that led to an overall improvement in the fit of the network to the data. We assessed whether a change improved the network model using the Bayesian Information Criterion (BIC)[[33](#_ENREF_33)]. This is equivalent to imposing a lower prior probability P(M) on models with larger numbers of parameters.

Even though edges in Bayesian networks are directed, in general we cannot infer causal relationships from the structure directly. For example, in a network with two nodes, $X^{1}$ and $X^{2}$, the two models $X^{1}\to X^{2}$ and $X^{2}\to X^{1}$ have equal probability distributions as $p(X^{1}\text{,}X^{2})=p(X^{2}\text{|}X^{1})p(X^{1})=p(X^{1}\text{|}X^{2})p(X^{2})$. Thus, using the data themselves, we cannot infer whether $X^{1}$ is causal of $X^{2}$, or vice versa. In a more general case, a network with three nodes, $X^{1}$, $X^{2}$, and $X^{3}$ , there are multiple groups of structures that are mathematically equivalent. For example, the following three different models, $\text{M}1: X^{1}\to X^{2}, X^{2}\to X^{3}$, $\text{M2}: X^{2}\to X^{1}, X^{2}\to X^{3}$, and $\text{M3}: X^{2}\to X^{1}, X^{3}\to X^{2}$, are Markov equivalent (which means that they all encode for the same conditional independent relationships). In the above case, all three structures encode the same conditional independent relationship, , $X^{1}$ and $X^{3}$ are independent conditioning on $X^{2}$, and they are mathematically equal

$$\begin{matrix} p(X) =p(\text{M}1|D)=p(X^{2}|X^{1})p(X^{1})p(X^{3}|X^{2}) \\ =p(\text{M2}|D)=p(X^{1}|X^{2})p(X^{2})p(X^{3}|X^{2}) \\ =p(\text{M3}|D)=p(X^{2}|X^{3})p(X^{3})p(X^{1}|X^{2}) \end{matrix}$$

Thus, we cannot infer whether $X^{1}$ is causal to $X^{2}$ or vice versa from these types of structures. However, there is a class of structures, V-shape structure (eg. $\text{Mv}: X^{1}\to X^{2}, X^{3}\to X^{2}$), which has no Markov equivalent structure. In this case, we can infer causal relationships. There are more parameters to estimate in the Mv model than M1, M2, or M3, which means a large penalty in BIC score for the Mv model. In practice, a large sample size is needed to differentiate the Mv model from the M1, M2, or M3 models.

Searching optimal Bayesian network structures given a dataset is a [non-deterministic polynomial-time](http://en.wikipedia.org/wiki/NP_%28complexity%29) hard. We employed a Monte Carlo Markov Chain method to do local searches of optimal structures. As the method is stochastic, the resulting structure will be different from each run. In this study, 1000 Bayesian networks were reconstructed using different random seeds to start the stochastic reconstruction process. From the resulting set of 1000 networks generated by this process, edges that appeared in greater than 30% of the networks were used to define a consensus network. The 30% cutoff threshold for edge inclusion is based on our simulation study[[34](#_ENREF_34)], where a 30% cutoff yielded the best tradeoff between recall rate and precision. The consensus network resulting by averaging may not be a Bayesian network (a directed acyclic graph). To make the consensus network structure into a directed acyclic graph, edges in this consensus network were removed if and only if 1) the edge was involved in a loop, and 2) the edge was the most weakly supported of all edges making up the loop. In the current study, we constructed Bayesian networks for each individual cohort as well as the combined dataset. Because genetic information was used to differentiate Markov equivalent structures, directed links in the Bayesian networks not only represent interactions among genes, but also can represent causal associations between genes. Therefore, these probabilistic networks allow us to predict the system's response to perturbations based on the identified relationships among genes.

**Assembling of asthma candidate gene list**

We generated a list on canonical asthma genes captured in gene network bioinformatics databases: OMIM[[35](#_ENREF_35)], Ingenuity[[36](#_ENREF_36)] and Metacore[[37](#_ENREF_37)] with genes that are associated with the key words: asthma, airway sensitivity, airway remodeling and airway hyper-sensitivity. From this search we arrived at 280 candidate genes that were also measured in the array we used in this study (**Table S5**).

**Epithelial cultures**

Healthy human donor lungs not suitable for transplantation and donated for medical research were obtained though the International Institute for the Advancement of Medicine (Edison, NJ). The study was approved (#H0-50110) by the ethics committees of the University of British Columbia. Primary airway epithelial cells were extracted by protease digestion of human airways as described previously[[38](#_ENREF_38)]. Briefly, the airways to the 3^rd^ generation were dissected and then rinsed with cold PBS without calcium and magnesium three times to remove blood and mucus. The epithelial layer lining 2-4 cm segments of trachea and bronchi was dissociated at 4^o^C for 16 hours in 100ml of MEM (Fisher Scientific) containing 1.4mg/ml Pronase and 0.1mg/ml of DNase (Roche Diagnostics). Dissociated epithelial cells were strained through a 70µm nylon mesh (Becton-Dickinson). Cells were then re-suspended and incubated in MEM supplemented with 10% FBS for 10 minutes and washed twice with MEM by centrifugation at 4^o^C to neutralize the pronase. Cells were then plated in tissue culture flasks and incubated at 37^o^C in a humidified 5% CO_2_ atmosphere in Bronchial Epithelial Growth Media (BEGM) (Cambrex, ON).

**Epithelial gene expression**

Airway epithelial cells at passage 1 were plated into 6-well plates at 10^5^ cells per well and cultured until 80-90% confluent. Culture supernatants were removed and total RNA was subsequently isolated using RNeasy Plus Mini-Kits (QIAGEN, Valencia, CA). Purified RNA was reverse transcribed into cDNA using Taqman® Reverse Transcription Reagents, and gene expression levels for *GSDMA* and *GSDMB* were determined by pre-developed Taqman® Gene Expression Assays as per the manufacturer's instructions (Applied Biosystems, Foster City, CA). The expression of the genes of interest was normalized to the housekeeping gene *GNB2L1* giving ΔCT values. The calculation 2^-ΔCT^ gives a relative value for gene expression as previously described[[39](#_ENREF_39)].

**Epithelial protein expression**

Airway epithelial cells were plated into 6-well plates at 10^5^ cells per well and cultured until 80-90% confluent. Culture supernatants were removed and total cell protein was obtained using Cell Protein Extraction Buffer (Biosource, Camarillo, CA), quantified by DC protein assay (Bio-Rad, Hercules, CA) and protein was resolved by SDS-PAGE, and transferred to a nitrocellulose membrane (Fisher Scientific, ON). Membranes were blocked in Odyssey blocking buffer (LI-COR Biosciences, Lincoln, NE) and probed with rabbit anti-human antibodies for GSDMA (HPA023313), GSDMB (HPA023925) and β-tubulin (T8535, all Sigma-Aldrich, ON). Detection was performed with anti-rabbit IRDye 800Dx conjugated and anti-goat IRDye 700Dx conjugated antibodies (Rockland Immunochemicals, Gilbertsville, PA) and the Odyssey Infrared Imaging System (LI-COR Biosciences). The density of the bands was quantified in two infrared channels independently using Odyssey software 2.1 (LI-COR Biosciences). The data are presented as a protein/β-tubulin density ratio.

**Immunohistochemical staining**

Donor airway sections were deparaffinized, rehydrated and antigens were retrieved by autoclaving the sections in citrate target retrieval solution (Dako) for 15 minutes at 120^o^C and 30 psi. Endogenous peroxidase was quenched with 3% hydrogen peroxide for 20 minutes, non-specific binding was blocked with 10% goat serum. Slides were incubated overnight with antibodies against human GSDMA (1:40, HPA023313) or IgG isotype control (Santa Cruz) at 4^o^C in 5% goat serum. Subsequent to three washes in Tris-buffered saline (TBS), sections were incubated with goat anti-rabbit secondary antibody (1:100, Vector Labs) for 2hrs, followed by incubation with streptavidin-horse radish peroxidase (Dako) for 20 minutes. Visualized staining of interest was developed with brown chromogen 3,3-diaminobenzidine (Dako) and counterstained with hematoxylin (Sigma). Slides were then dehydrated and mounted with Cytoseal 60 (Richard-Allan Scientific).

**References**

1. (1995) Standardization of Spirometry, 1994 Update. American Thoracic Society. Am J Respir Crit Care Med 152: 1107-1136.

2. (1995) American Thoracic Society. Single-breath carbon monoxide diffusing capacity (transfer factor). Recommendations for a standard technique--1995 update. Am J Respir Crit Care Med 152: 2185-2198.

3. Rabe KF, Hurd S, Anzueto A, Barnes PJ, Buist SA, et al. (2007) Global strategy for the diagnosis, management, and prevention of chronic obstructive pulmonary disease: GOLD executive summary. Am J Respir Crit Care Med 176: 532-555.

4. Chen Y, Zhu J, Lum PY, Yang X, Pinto S, et al. (2008) Variations in DNA elucidate molecular networks that cause disease. Nature 452: 429-435.

5. Irizarry RA, Hobbs B, Collin F, Beazer-Barclay YD, Antonellis KJ, et al. (2003) Exploration, normalization, and summaries of high density oligonucleotide array probe level data. Biostatistics 4: 249-264.

6. Purcell S, Neale B, Todd-Brown K, Thomas L, Ferreira MA, et al. (2007) PLINK: a tool set for whole-genome association and population-based linkage analyses. Am J Hum Genet 81: 559-575.

7. Price AL, Patterson NJ, Plenge RM, Weinblatt ME, Shadick NA, et al. (2006) Principal components analysis corrects for stratification in genome-wide association studies. Nat Genet 38: 904-909.

8. Li Y, Willer CJ, Ding J, Scheet P, Abecasis GR (2010) MaCH: using sequence and genotype data to estimate haplotypes and unobserved genotypes. Genet Epidemiol 34: 816-834.

9. Schadt EE, Molony C, Chudin E, Hao K, Yang X, et al. (2008) Mapping the genetic architecture of gene expression in human liver. PLoS Biol 6: e107.

10. Zeggini E, Scott LJ, Saxena R, Voight BF, Marchini JL, et al. (2008) Meta-analysis of genome-wide association data and large-scale replication identifies additional susceptibility loci for type 2 diabetes. Nat Genet 40: 638-645.

11. Benovoy D, Kwan T, Majewski J (2008) Effect of polymorphisms within probe-target sequences on olignonucleotide microarray experiments. Nucleic Acids Res 36: 4417-4423.

12. Alberts R, Terpstra P, Li Y, Breitling R, Nap JP, et al. (2007) Sequence polymorphisms cause many false cis eQTLs. PLoS One 2: e622.

13. Hindorff LA, Sethupathy P, Junkins HA, Ramos EM, Mehta JP, et al. (2009) Potential etiologic and functional implications of genome-wide association loci for human diseases and traits. Proc Natl Acad Sci U S A 106: 9362-9367.

14. Choudhry S, Taub M, Mei R, Rodriguez-Santana J, Rodriguez-Cintron W, et al. (2008) Genome-wide screen for asthma in Puerto Ricans: evidence for association with 5q23 region. Hum Genet 123: 455-468.

15. DeWan AT, Triche EW, Xu X, Hsu LI, Zhao C, et al. (2010) PDE11A associations with asthma: results of a genome-wide association scan. J Allergy Clin Immunol 126: 871-873 e879.

16. Gudbjartsson DF, Bjornsdottir US, Halapi E, Helgadottir A, Sulem P, et al. (2009) Sequence variants affecting eosinophil numbers associate with asthma and myocardial infarction. Nat Genet 41: 342-347.

17. Hancock DB, Romieu I, Shi M, Sienra-Monge JJ, Wu H, et al. (2009) Genome-wide association study implicates chromosome 9q21.31 as a susceptibility locus for asthma in mexican children. PLoS Genet 5: e1000623.

18. Himes BE, Hunninghake GM, Baurley JW, Rafaels NM, Sleiman P, et al. (2009) Genome-wide association analysis identifies PDE4D as an asthma-susceptibility gene. Am J Hum Genet 84: 581-593.

19. Hui J, Oka A, James A, Palmer LJ, Musk AW, et al. (2008) A genome-wide association scan for asthma in a general Australian population. Hum Genet 123: 297-306.

20. Kim SH, Cho BY, Park CS, Shin ES, Cho EY, et al. (2009) Alpha-T-catenin (CTNNA3) gene was identified as a risk variant for toluene diisocyanate-induced asthma by genome-wide association analysis. Clin Exp Allergy 39: 203-212.

21. Mathias RA, Grant AV, Rafaels N, Hand T, Gao L, et al. (2010) A genome-wide association study on African-ancestry populations for asthma. J Allergy Clin Immunol 125: 336-346 e334.

22. Moffatt MF, Gut IG, Demenais F, Strachan DP, Bouzigon E, et al. (2010) A large-scale, consortium-based genomewide association study of asthma. N Engl J Med 363: 1211-1221.

23. Moffatt MF, Kabesch M, Liang L, Dixon AL, Strachan D, et al. (2007) Genetic variants regulating ORMDL3 expression contribute to the risk of childhood asthma. Nature 448: 470-473.

24. Ober C, Tan Z, Sun Y, Possick JD, Pan L, et al. (2008) Effect of variation in CHI3L1 on serum YKL-40 level, risk of asthma, and lung function. N Engl J Med 358: 1682-1691.

25. Sleiman PM, Flory J, Imielinski M, Bradfield JP, Annaiah K, et al. (2010) Variants of DENND1B associated with asthma in children. N Engl J Med 362: 36-44.

26. Weidinger S, Gieger C, Rodriguez E, Baurecht H, Mempel M, et al. (2008) Genome-wide scan on total serum IgE levels identifies FCER1A as novel susceptibility locus. PLoS Genet 4: e1000166.

27. Storey JD, Tibshirani R (2003) Statistical significance for genomewide studies. Proc Natl Acad Sci U S A 100: 9440-9445.

28. Schadt EE, Lamb J, Yang X, Zhu J, Edwards S, et al. (2005) An integrative genomics approach to infer causal associations between gene expression and disease. Nat Genet 37: 710-717.

29. Ravasz E, Somera AL, Mongru DA, Oltvai ZN, Barabasi AL (2002) Hierarchical organization of modularity in metabolic networks. Science 297: 1551-1555.

30. Langfelder P, Zhang B, Horvath S (2008) Defining clusters from a hierarchical cluster tree: the Dynamic Tree Cut package for R. Bioinformatics 24: 719-720.

31. Pearl J (1988) Probabilistic reasoning in intelligent systems : networks of plausible inference. San Mateo, Calif.: Morgan Kaufmann Publishers. 552 p.

32. Madigan DaY J (1995) Bayesian graphical models for discrete data. International Statistical Review 63: 215-232.

33. Schwarz G (1978) Estimating the dimension of a model. Annals of Statistics 6: 461-464.

34. Zhu J, Wiener MC, Zhang C, Fridman A, Minch E, et al. (2007) Increasing the power to detect causal associations by combining genotypic and expression data in segregating populations. PLoS Comput Biol 3: e69.

35. Hamosh A, Scott AF, Amberger J, Valle D, McKusick VA (2000) Online Mendelian Inheritance in Man (OMIM). Hum Mutat 15: 57-61.

36. Calvano SE, Xiao W, Richards DR, Felciano RM, Baker HV, et al. (2005) A network-based analysis of systemic inflammation in humans. Nature 437: 1032-1037.

37. Ekins S, Nikolsky Y, Bugrim A, Kirillov E, Nikolskaya T (2007) Pathway mapping tools for analysis of high content data. Methods Mol Biol 356: 319-350.

38. Hackett TL, Shaheen F, Johnson A, Wadsworth S, Pechkovsky DV, et al. (2008) Characterization of side population cells from human airway epithelium. Stem Cells 26: 2576-2585.

39. Hackett TL, Warner SM, Stefanowicz D, Shaheen F, Pechkovsky DV, et al. (2009) Induction of epithelial-mesenchymal transition in primary airway epithelial cells from patients with asthma by transforming growth factor-beta1. Am J Respir Crit Care Med 180: 122-133.
